# The Circumstance-Driven Bivariate Integer-Valued Autoregressive Model

**DOI:** 10.3390/e26020168

**Published:** 2024-02-15

**Authors:** Huiqiao Wang, Christian H. Weiß

**Affiliations:** 1 Department of Mathematics and Statistics, Helmut Schmidt University, Holstenhofweg 85, 22043 Hamburg, Germany; weissc@hsu-hh.de; 2 Department of Statistics, Southwestern University of Finance and Economics, Chengdu 611130, China

**Keywords:** CuBINAR model, non-stationarity, circumstance driven

## Abstract

The novel circumstance-driven bivariate integer-valued autoregressive (CuBINAR) model for non-stationary count time series is proposed. The non-stationarity of the bivariate count process is defined by a joint categorical sequence, which expresses the current state of the process. Additional cross-dependence can be generated via cross-dependent innovations. The model can also be equipped with a marginal bivariate Poisson distribution to make it suitable for low-count time series. Important stochastic properties of the new model are derived. The Yule–Walker and conditional maximum likelihood method are adopted to estimate the unknown parameters. The consistency of these estimators is established, and their finite-sample performance is investigated by a simulation study. The scope and application of the model are illustrated by a real-world data example on sales counts, where a soap product in different stores with a common circumstance factor is investigated.

## 1. Introduction

Integer-valued time series data are encountered in many fields in practice, such as epidemiology, insurance, finance, and quality control (see [1] for a comprehensive survey). There are many approaches to model such count data. One pioneering approach is to use a random thinning operator as a substitute of the multiplication in the traditional autoregressive (AR) model to construct an integer-valued autoregressive (INAR) model (see [2,3]). The first-order INAR (INAR(1)) model is defined as follows:

(1) $$\begin{array}{c} X_{t}=\alpha \circ X_{t-1}+\varepsilon_{t}, \end{array}$$

$t\in N_{+}$, where $\alpha \circ X_{t-1}$ is defined as $\alpha \circ X_{t-1}=\sum_{i=1}^{X_{t-1}}Y_{i}$, with $\left\{Y_{i}\right\}$ being a sequence of independent and identically distributed (i. i. d.) Bernoulli random variables with parameter α∈[0,1). The innovations $\varepsilon_{t}$ are i. i. d. count random variables, i.e., having the range $N_{0}=\left\{0,1,\ldots \right\}$, where the default choice is a Poisson distribution for $\varepsilon_{t}$ [3]. Many researchers have generalized the basic INAR(1) model to better fit real data. To handle overdispersion or zero-inflation features in data, the negative-binomial, geometric or zero-inflated Poisson distribution have been proposed to replace the Poisson distribution of innovation term $\varepsilon_{t}$ (see [1] for details and references). Also, different types of thinning operator have been proposed in the literature (see [4] for a survey). Other proposals generalize the model from the view of the model structure. Thyregod et al. [5] first proposed the self-exciting threshold (SET) integer-valued model, which is also studied in [6]. A comprehensive introduction to SET-INAR models can be found in [7].

The aforementioned articles focus on stationary count time series. To handle the non-stationary case, ref. [8] applied the difference method to non-stationary count data and introduced the signed binomial thinning operator to allow for negative values after differencing. Nastić et al. [9] constructed the random-environment INAR(1) model to characterize non-stationarity in integer-valued time series, where the parameters in the model are influenced by different states of the environment, the evolution of which is defined through a selection mechanism from a Markov chain. Laketa et al. [10] generalized this work to a *p*-th order model.

Nowadays, there is increasing interest in multivariate integer-valued time series models, where most contributions focus on the bivariate case. Such types of data are commonly encountered in real-world applications. For example, ref. [11] consider the number of daytime and nighttime accidents in a certain area, which are at distinct levels but present cross-correlation due to the same road conditions. Latour [12] first proposed a general multivariate INAR(1) model and proved the existence and relevant properties of the model. Further model properties have been studied by [13]. The bivariate INAR(1) model introduced by [11] is defined as

(2) $$\begin{array}{c} X_{t}=A\circ X_{t-1}+R_{t}=\left[\begin{array}{cc} \alpha_{1} & 0\\ 0 & \alpha_{2} \end{array}\right]\circ \left[\begin{array}{c} X_{1,t-1}\\ X_{2,t-1} \end{array}\right]+\left[\begin{array}{c} \varepsilon_{1,t}\\ \varepsilon_{2,t} \end{array}\right], \end{array}$$

where “∘” is the binomial thinning operator defined as in (1), with $\alpha_{1},\alpha_{2}\in \left[0,1\right)$. $\varepsilon_{i,t}$ is the innovation term of the *i*-th series, i=1,2. The role of the A∘ is the usual matrix multiplication, and it also keeps the properties of the binomial thinning operation. Regarding further research on bivariate INAR(1) models, we refer to the work of [14,15], while we refer to the work of [16,17,18] for research on bivariate integer-valued moving average (INMA(1)) models. In addition, ref. [19] introduced a bivariate model for integer-valued time series with a finite range of counts. Yu et al. [20] introduced the new bivariate random-coefficient integer-valued autoregressive (BRCINAR(1)) model to allow the coefficients to be random. Although the present article concentrates on thinning-based models for count time series (which allow us to specify the marginal distribution of $\left(X_{t}\right)$), it should also be briefly mentioned that different approaches have been proposed in the literature. Regression-type models for (bivariate) count time series (where the conditional distributions of $X_{t}|X_{t-1},\ldots$ are specified rather than the marginal ones) have been proposed by, e.g., [21,22,23]. In contrast, ref. [24] derive a multivariate count time series model with Poisson marginal distributions from underlying multivariate Gaussian time series.

Non-stationarity is an important feature of real-world time series data, whether for one-dimensional or multi-dimensional data. The change in external factors may change the structure or level in the data. There has not been much work to explore the non-stationarity of bivariate count time series. One main problem is the distribution of the model, which becomes complicated with increasing dimension. We propose a new model to characterize the non-stationarity in bivariate integer-valued time series. Inspired by [9,25], we suppose the parameters in the model to be affected by the different states of the circumstance, to characterize the intrinsic nature of non-stationarity in the data. In contrast to [25], the novel model is able to incorporate additional cross-dependence, and it is also suitable for low-count time series having a bivariate Poisson marginal distribution (see Remark 3 for further details). In Section 2, we propose the new first-order circumstance-driven bivariate INAR (CuBINAR(1)) model, and establish its stochastic properties. Estimation methods and their asymptotic properties are discussed in Section 3. In Section 4, the performance of the estimators is evaluated by a simulation study. A real-data application is presented in Section 5. Summary and conclusions are given in Section 6.

## 2. Model Construction

In this section, we introduce the new non-stationary CuBINAR(1) model, where the bivariate count random variable $X_{t}$ at time *t* is not only influenced by $X_{t-1}$, but also by the underlying circumstance state $s_{t}$ as we define in (3).

**Definition** **1.** *The CuBINAR(1) process $\left(X_{t}\right)$ with range $N_{0}^{2}$ is defined by the recursive scheme*

(3) $$\begin{array}{c} X_{t}\left(s_{t}\right)=A\circ X_{t-1}\left(s_{t-1}\right)+\varepsilon_{t}\left(s_{t},s_{t-1}\right). \end{array}$$

*The model can be rewritten in matrix form as*

$$\left[\begin{array}{c} X_{1,t}\left(s_{t}\right)\\ X_{2,t}\left(s_{t}\right) \end{array}\right]=\left[\begin{array}{cc} \alpha_{1} & 0\\ 0 & \alpha_{2} \end{array}\right]\circ \left[\begin{array}{c} X_{1,t-1}\left(s_{t-1}\right)\\ X_{2,t-1}\left(s_{t-1}\right) \end{array}\right]+\left[\begin{array}{c} \varepsilon_{1,t}\left(s_{t},s_{t-1}\right)\\ \varepsilon_{2,t}\left(s_{t},s_{t-1}\right) \end{array}\right],$$

*i.e., the i-th component of the vector $X_{t}\left(s_{t}\right)$ satisfies*

$$\begin{array}{c} X_{i,t}\left(s_{t}\right)=\alpha_{i}\circ X_{i,t}\left(s_{t-1}\right)+\varepsilon_{i,t}\left(s_{t},s_{t-1}\right),\;i=1,2,\;t=2,\ldots ,n. \end{array}$$

*Here, $s_{t}$ represents the state at time t with possible values in S={1,2,…,S}, where S⩾2 is total number of states. $X_{i,t}\left(s_{t}\right)$ is the t-th observation of the i-th series depending on the state $s_{t}$, and $\varepsilon_{i,t}\left(s_{t},s_{t-1}\right)$ is the corresponding innovation term depending on the states $s_{t}$ and $s_{t-1}$. In addition, $\varepsilon_{t}\left(s_{t},s_{t-1}\right)$ is independent of $A\circ X_{t-1}\left(s_{t-1}\right)$ and $X_{k}\left(s_{k}\right)$ for k<t, where the definition of the binomial thinning operator “∘” is given after (1).*

**Remark** **1.** *We assume the different states of the circumstance are already realized. In the simulation part, we first need to generate the sample path of the states. To characterize the variation of the states, we adopt a Markov chain to generate it: given the initial probability vector $p_{0}=\left(p_{1},\ldots ,p_{S}\right)$ and transition matrix $P=\left(\begin{array}{ccc} p_{11} & \cdots & p_{1S}\\ \vdots & \ddots & \vdots \\ p_{S1} & \cdots & p_{SS} \end{array}\right)$, the sample path of the states can be obtained. In real-data analysis, we first need to know the states of the observations. In the data example discussed in Section 5, the sequence of states is defined according to a possible sales promotion.*

In the subsequent Proposition 1, we introduce the important special case of a CuBINAR(1) process having a bivariate Poisson marginal distribution (thus abbreviated as Poi-CuBINAR(1)). More precisely, $\left(X_{t}\right)$ is said to follow the Poi-CuBINAR(1) model if $\left\{X_{1,t},X_{2,t}\right\}∼\mathrm{BPoi}\left(\lambda_{1}\left(s_{t}\right),\lambda_{2}\left(s_{t}\right),\phi \right)$ for appropriately chosen parameter values (see Remark 2 below). Here, we use the same definition of the BPoi-distribution as in [11,13], i.e., the parameters of $X∼\mathrm{BPoi}(\lambda_{1},\lambda_{2},\phi )$ are defined as the mean of $X_{1}$, mean of $X_{2}$, and covariance between $X_{1},X_{2}$, respectively. So the probability generating function (PGF) of X would be given by

$$E\left[a_{1}^{X_{1}}\cdot a_{2}^{X_{2}}\right]=\exp\left\{\lambda_{1}\left(a_{1}-1\right)+\lambda_{2}\left(a_{2}-1\right)+\phi \left(a_{1}-1\right)\left(a_{2}-1\right)\right\}.$$

It shall be shown that, in analogy to the univariate Poi-INAR(1) model [4], BPoi-observations are achieved by assuming BPoi-innovations.

**Proposition** **1.** *Let $\left(X_{t}\right)$ be a CuBINAR(1) process according to Definition 1. Then, $\left(X_{t}\right)$ constitutes a Poi-CuBINAR(1) process with $\left\{X_{1,t},X_{2,t}\right\}∼\mathrm{BPoi}\left(\lambda_{1}\left(s_{t}\right),\lambda_{2}\left(s_{t}\right),\phi \right)$ if the distribution of the model’s innovation term satisfies*

$$\left\{\varepsilon_{1,t}\left(s_{t},s_{t-1}\right),\varepsilon_{2,t}\left(s_{t},s_{t-1}\right)\right\}∼\mathrm{BPoi}\left(\lambda_{1}\left(s_{t}\right)-\lambda_{1}\left(s_{t-1}\right)\alpha_{1},\lambda_{2}\left(s_{t}\right)-\lambda_{2}\left(s_{t-1}\right)\alpha_{2},\phi^{*}\right),$$

where $\phi^{*}=\phi \;\left(1-\alpha_{1}\alpha_{2}\right)$.

For the detailed proof, we refer to Appendix B. Note that for the derivation of Proposition 1, it is crucial that A is a diagonal matrix. While Definition 1 could generally be extended to a non-diagonal A, we would lose the marginal BPoi-property (see also [13] for analogous results in the stationary case). In fact, the components would then not follow univariate INAR(1) models any more.

**Remark** **2.** *We must ensure the parameters of the $\mathrm{BPoi}(\lambda_{1}\left(s_{t}\right),\lambda_{2}\left(s_{t}\right),\phi )$-distribution in Proposition 1 are truly positive, i.e., $\lambda_{i}\left(s\right)-\lambda_{i}\left(r\right)\alpha_{i}-\phi +\phi \alpha_{1}\alpha_{2}>0$ holds for i=1,2 and r,s∈S at same time, where s and r are the realizations of $s_{t}$ and $s_{t-1}$, respectively. Hence, there are $2\cdot S^{2}$ inequalities that need to be satisfied simultaneously.*

**Remark** **3.** *As already indicated in Section 1, the novel CuBINAR(1) model is constructed in a similar way as the bivariate “random environment INAR(1) model” proposed by [25], referred to as RE-BINAR(1) hereafter. But, there are also noteworthy differences between these two models. First, for the BRrNGINAR(1) model of [25], cross-correlation between the two series is solely caused by the common state while their innovation sequences are mutually independent. Our CuBINAR(1) model, by contrast, allows for additional cross-correlation being caused by the cross-correlated innovation term. For example, in case of the Poi-CuBINAR(1) model, the innovation term $\left\{\varepsilon_{1,t}\left(s_{t},s_{t-1}\right),\varepsilon_{2,t}\left(s_{t},s_{t-1}\right)\right\}$ stems from a bivariate Poisson distribution, also leading to a bivariate Poisson distribution for $\left\{X_{1,t}\left(s_{t}\right),X_{2,t}\left(s_{t}\right)\right\}$ (see Proposition 1). Then, choosing ϕ>0 leads to additional cross-correlation, while mutually independent innovations series are included as the special case ϕ=0. Altogether, the user has more flexibility to fit the model to given time series data.*

*Second, ref. [25] construct their model based on the negative-binomial thinning operator and geometric marginal distributions, so the model is particularly useful for overdispersed counts. Our CuBINAR(1) model, by contrast, uses binomial thinnings. As discussed by [4], binomial thinnings can also be used to generate common overdispersed marginal distributions (including the geometric one). But, in addition, the equidispersed Poisson distribution is also possible, as it is often observed for low-counts time series. In the special case of the Poi-CuBINAR(1) model introduced in Proposition 1, the process is equipped with a marginal bivariate Poisson distribution. Altogether, we believe that our novel CuBINAR(1) model constitutes a valuable complement to existing models for non-stationary bivariate count time series.*

The following proposition provides some (conditional) moment properties of the Poi-CuBINAR(1) model, which shall useful to obtain the Yule–Walker estimators.

**Proposition** **2.** *Let $\left(X_{t}\right)$ be the Poi-CuBINAR(1) process according to Proposition 1. Let us denote the means of $X_{i,t}\left(s_{t}\right)$, $X_{i,t-1}\left(s_{t-1}\right)$, and $\varepsilon_{i,t}\left(s_{t},s_{t-1}\right)$ as $\lambda_{i}\left(s_{t}\right)$, $\lambda_{i}\left(s_{t-1}\right)$, and $\mu_{i}\left(s_{t},s_{t-1}\right)$, respectively, for i=1,2. Then, the following assertions hold:*

*(i)*  *$E\left[X_{i,t}\left(s_{t}\right)|X_{i,t-1}\left(s_{t-1}\right)\right]=\alpha_{i}\cdot X_{i,t-1}\left(s_{t-1}\right)+\mu_{i}\left(s_{t},s_{t-1}\right)$;*
*$E\left[X_{i,t}\left(s_{t}\right)\right]=\lambda_{i}\left(s_{t}\right)$;*
*(ii)*  *$Var\left(X_{i,t}\left(s_{t}\right)\right)=\lambda_{i}\left(s_{t}\right)$;*
*$cov\left(X_{i,t}\left(s_{t}\right),X_{i,t-1}\left(s_{t-1}\right)\right)=\alpha_{i}\cdot \lambda_{i}\left(s_{t-1}\right)$;*
*(iii)*  *$cov(X_{1,t}\left(s_{t}\right),X_{2,t}\left(s_{t}\right))=\phi$;*
*$cov\left(X_{1,t}\left(s_{t}\right),X_{2,t-k}\left(s_{t-k}\right)\right)=\alpha_{1}^{k}\cdot \phi$.*

For the proof of Proposition 2, see Appendix C.

## 3. Parameter Estimation

In this section, we consider the Yule–Walker (YW) method and the conditional maximum likelihood (CML) method to estimate the parameter values of the Poi-CuBINAR(1) model.

### 3.1. Yule–Walker Estimation

From now on, let us use the following notations, for i=1,2 and r,s∈S:

(4) $$\begin{array}{cc} \mu_{i}\left(s\right) & =E[X_{i,t}\left(s_{t}\right)|s_{t}=s],\\ \gamma_{ii,0}\left(s\right) & =Var(X_{it}\left(s_{t}\right)|s_{t}=s),\\ \gamma_{12,0}\left(s\right) & =cov(X_{1,t}\left(s_{t}\right),X_{2,t}\left(s_{t}\right)|s_{t}=s),\\ \gamma_{i}\left(r,s\right) & =cov(X_{i,t}\left(s_{t}\right),X_{i,t-1}\left(s_{t-1}\right)|s_{t-1}=r,s_{t}=s). \end{array}$$

For the Poi-CuBINAR(1) model, the $X_{i,t}$ are Poisson-distributed, so $\mu_{i}\left(s\right)$ is equal to $\gamma_{ii,0}\left(s\right)$.

Given the realized states, the corresponding sample moments are as follows:

(5) $$\begin{array}{cc} {\hat{\mu }}_{i}\left(s\right) & =\frac{1}{n_{s}}\sum_{t=1}^{n}X_{i,t}\left(s_{t}\right)1_{\left\{s_{t}=s\right\}}, \end{array}$$

(6) $$\begin{array}{cc} {\hat{\gamma }}_{ij,0}\left(s\right) & =\frac{1}{n_{s}}\sum_{t=1}^{n}\left(X_{i,t}\left(s_{t}\right)-{\hat{\mu }}_{i}\left(s\right)\right)\left(X_{j,t}\left(s_{t}\right)-{\hat{\mu }}_{j}\left(s\right)\right)1_{\left\{s_{t}=s\right\}}, \end{array}$$

(7) $$\begin{array}{cc} {\hat{\gamma }}_{i}\left(r,s\right) & =\frac{1}{n_{r,s}}\sum_{t=2}^{n}\left(X_{i,t}\left(s_{t}\right)-{\hat{\mu }}_{i}\left(s\right)\right)\left(X_{i,t-1}\left(s_{t-1}\right)-{\hat{\mu }}_{i}\left(r\right)\right)1_{\left\{s_{t-1}=r,s_{t}=s\right\}}. \end{array}$$

In Equation (6), i=j leads to ${\hat{\gamma }}_{ii,0}\left(s\right)$, which is the empirical conditional variance given the state *s*, and which estimates $\gamma_{ii,0}\left(s\right)$. Otherwise, it equals the empirical conditional cross-covariance and thus estimates $\gamma_{12,0}\left(s\right)$. $n_{s}=\sum_{t=1}^{n}1_{\left\{s_{t}=s\right\}}$ is the sample size under state *s*, $n_{r,s}=\sum_{t=2}^{n}1_{\left\{s_{t-1}=r,s_{t}=s\right\}}$ the one under the condition that the state at *t* equals *s* and that at t−1 equals *r*. $1_{A}$ denotes the indicator function, which is equal to 1 (0) if *A* is true (false).

**Remark** **4.** *The parameters ϕ and $\alpha_{i}$ can be expressed by the following equations:*

(8) $$\begin{array}{cc} \phi & =\sum_{s=1}^{S}\frac{n_{s}}{n}\cdot \gamma_{12,0}\left(s\right), \end{array}$$

(9) $$\begin{array}{cc} \alpha_{i} & =\sum_{r=1}^{S}\sum_{s=1}^{S}\frac{n_{r,s}}{n}\cdot \frac{\gamma_{i}\left(r,s\right)}{\gamma_{ii,0}\left(r\right)}, \end{array}$$

*see Appendix D for the proof. Equations (8) and (9) can be used to define estimators of ϕ and $\alpha_{i}$, respectively.*

Following Remark 4, we define the Yule–Walker estimators as follows:

(10) $$\begin{array}{cc} {\hat{\alpha }}_{i}^{yw} & =\sum_{r=1}^{S}\sum_{s=1}^{S}\frac{n_{r,s}}{n-1}\frac{{\hat{\gamma }}_{i}\left(r,s\right)}{{\hat{\gamma }}_{ii,0}\left(r\right)}, \end{array}$$

(11) $$\begin{array}{cc} {\hat{\lambda }}_{i}{\left(s\right)}^{yw} & ={\hat{\mu }}_{i}\left(s\right),\;s\in S, \end{array}$$

(12) $$\begin{array}{cc} {\hat{\phi }}^{yw} & =\sum_{s=1}^{S}\frac{n_{s}}{n}{\hat{\gamma }}_{12,0}\left(s\right). \end{array}$$

In next theorem, we prove that these Yule–Walker estimators are consistent.

**Theorem** **1.** *The Yule–Walker estimators ${\hat{\alpha }}_{i}^{yw}$, ${\hat{\lambda }}_{i}{\left(s\right)}^{yw}$ and ${\hat{\phi }}^{yw}$, i=1,2, s∈S defined in Equations (10)–(12) are consistent.*

The proof of Theorem 1 is provided by Appendix E.

### 3.2. Conditional Maximum Likelihood Estimation

From the first-order Markov property of the CuBINAR(1) model according to Definition 1, the conditional log-likelihood function is expressed as

$$\begin{array}{c} L=\sum_{t=2}^{n}\log P\left(X_{t}\left(s_{t}\right)|X_{t-1}\left(s_{t-1}\right)\right), \end{array}$$

where $P(X_{t}\left(s_{t}\right)|X_{t-1}\left(s_{t-1}\right))$ is the transition probability given the realized states. It has the following expression, where, for simplicity, we omit the states $s_{t},s_{t-1}$ in parentheses after $X_{t},X_{t-1}$:

(13) P(Xt∣Xt−1)=P(X1,t=x1,t,X2,t=x2,t∣X1,t−1=x1,t−1,X2,t−1=x2,t−1)=∑k=0min(x1,t,x1,t−1)∑l=0min(x2,t,x2,t−1)x1,t−1k·α1k·(1−α1)x1,t−1−k·x2,t−1l·α2l·(1−α2)x2,t−1−l·f(x1,t−k,x2,t−l),

where $f\left(x_{1,t}-k,x_{2,t}-l\right)=e^{-(\lambda_{1}^{*}+\lambda_{2}^{*}+\phi^{*})}\sum_{i=0}^{\min(x_{1,t}-k,x_{2,t}-l)}\frac{{\lambda_{1}^{*}}^{\left(x_{1,t}-k-i\right)}{\lambda_{2}^{*}}^{\left(x_{2,t}-l-i\right)}{\phi^{*}}^{i}}{\left(x_{1,t}-k-i\right)!\left(x_{2,t}-l-i\right)!i!}$. Here, $\lambda_{1}^{*}=\lambda_{1}\left(s_{t}\right)-\lambda_{1}\left(s_{t-1}\right)\alpha_{1}-\phi^{*}$, $\lambda_{2}^{*}=\lambda_{2}\left(s_{t}\right)-\lambda_{2}\left(s_{t-1}\right)\alpha_{2}-\phi^{*}$, and $\phi^{*}=\phi -\phi \alpha_{1}\alpha_{2}$. The CML estimates are computed by applying a numerical optimization routine to the log-likelihood function L. Based on the inverse of the numerical Hessian, one can then calculate approximate standard errors (see Remark B.2.1.2 in [1] for details.)

## 4. Simulation Study

In this section, we conduct a simulation study to evaluate the performance of the YW and CML estimators. The standard errors and biases of the estimators are calculated based on 10,000 replications, where the sample sizes are n∈{300,900,1500,2100}. Since we assume that the states of the circumstance are already realized, we first need to generate the circumstance states for each replication run, which is performed via the Markov chain approach described in Remark 1. In order to implement this, the initial probability vector and the transition probability matrix need to be specified.

Two different scenarios are considered. In the first scenario, we assume that the observations are driven by three different states of the circumstance, in which case we also consider two different types of transition probability matrix: one with initial probability vector $p_{0}^{\left(1\right)}=\left(0.33,0.33,0.34\right)$ and transition probability matrix $\left(\begin{array}{ccc} 0.4 & 0.3 & 0.3\\ 0.3 & 0.4 & 0.3\\ 0.3 & 0.3 & 0.4 \end{array}\right)$, the other with $p_{0}^{\left(2\right)}=\left(0.6,0.3,0.1\right)$ and $\left(\begin{array}{ccc} 0.6 & 0.3 & 0.1\\ 0.1 & 0.6 & 0.3\\ 0.3 & 0.1 & 0.6 \end{array}\right)$. Furthermore, three different parameter groups are considered:

(a) $\left(\alpha_{1},\alpha_{2},\phi ,\phi^{*},\lambda_{1}\left(1\right),\lambda_{1}\left(2\right),\lambda_{1}\left(3\right),\lambda_{2}\left(1\right),\lambda_{2}\left(2\right),\lambda_{3}\left(3\right)\right)$
  =(0.15,0.2,0.5,0.485,1,2,3,4,5,6);
(b) $\left(\alpha_{1},\alpha_{2},\phi ,\phi^{*},\lambda_{1}\left(1\right),\lambda_{1}\left(2\right),\lambda_{1}\left(3\right),\lambda_{2}\left(1\right),\lambda_{2}\left(2\right),\lambda_{3}\left(3\right)\right)$
  =(0.15,0.5,0.5,0.4625,1,2,3,4,5,6);
(c) $\left(\alpha_{1},\alpha_{2},\phi ,\phi^{*},\lambda_{1}\left(1\right),\lambda_{1}\left(2\right),\lambda_{1}\left(3\right),\lambda_{2}\left(1\right),\lambda_{2}\left(2\right),\lambda_{3}\left(3\right)\right)$
  =(0.4,0.25,1,0.9,3,4,5,2,3,4).

In the second scenario, we suppose the circumstance to only have two states. Two different transition probability matrices are set, namely $\left(\begin{array}{cc} 0.4 & 0.6\\ 0.6 & 0.4 \end{array}\right)$ and $\left(\begin{array}{cc} 0.8 & 0.2\\ 0.2 & 0.8 \end{array}\right)$, respectively, and the corresponding initial probability vectors are (0.5,0.5) and (0.3,0.7).

(d)
$\left(\alpha_{1},\alpha_{2},\phi ,\phi^{*},\lambda_{1}\left(s_{1}\right),\lambda_{1}\left(s_{2}\right),\lambda_{2}\left(s_{1}\right),\lambda_{2}\left(s_{2}\right)\right)$

  =(0.15,0.2,0.5,0.4625,1,3,3,5);
(e)
$\left(\alpha_{1},\alpha_{2},\phi ,\phi^{*},\lambda_{1}\left(s_{1}\right),\lambda_{1}\left(s_{2}\right),\lambda_{2}\left(s_{1}\right),\lambda_{2}\left(s_{2}\right)\right)$

  =(0.25,0.5,1,0.875,2,4,5,7).

Note that the $\alpha_{1},\alpha_{2}$ in all parameters groups satisfy the constraints in Remark 2.

The estimation results of the YW and CML estimators are presented in Table A1, Table A2, Table A3, Table A4, Table A5, Table A6, Table A7, Table A8, Table A9 and Table A10 in Appendix A. It can be seen that if the sample size increases, all estimates converge to the true parameter values: the standard errors and biases decrease towards to 0, confirming the consistency of the estimators. Comparing the finite-sample properties among the YW and CML approaches, it becomes clear that the additional computational effort required for CML also leads to an improved performance. The CML estimates are less biased and less dispersed, where the additional gain in performance is particularly large for the dependence parameters $\alpha_{1}$, $\alpha_{2}$, and ϕ. So if possible, the CML approach should be preferred for parameter estimation.

## 5. A Real-Data Example

In this section, we analyze data referring to the number of sold items of a soap product (category “wsoa” in Dominick’s Data (https://www.chicagobooth.edu/research/kilts/datasets/dominicks, accessed on 10 November 2021) from the James M. Kilts Center, University of Chicago, Booth School of Business), which are counted on a weekly basis. We focus on the product “Level 200 Bath 6 BA” (code number 1111132012) in the soap category, and we consider the bivariate count time series for stores 54 and 88 in the period April 14, 1994, to 4 May 1995 (weeks 240–295, n=56). The movement files also provide information on a sales promotion for the product. There are three types of promotion (labeled ‘B’, ‘C’ and ‘S’), which we summarized into one category, namely “sales promotion—yes or no” (yes: state 1; no: state 2). As the number of sold items might be affected by whether the product is under promotion or not, the action of promotion can be seen as a potential circumstance-driving factor. It is also worth mentioning that ref. [26] analyzed count data from the soap category (product “Zest White Water”), but using a Hidden-Markov model instead (i.e., they did not utilize the information about sales promotion).

**Figure 1:**
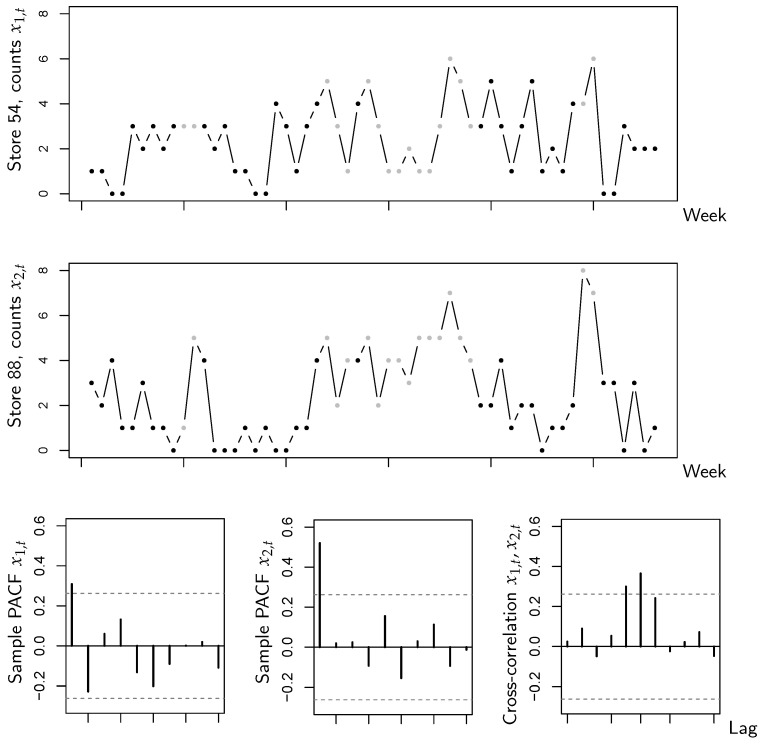
Bivariate sales counts from Section 5: time series plots, sample PACFs, and cross-correlations of both sub-series. The dots in the time series plots are printed in gray (black) color if the state equals 1 (2).

The data are shown in Figure 1, with the counts in state 1 being plotted in gray color. The PACFs indicate an AR(1)-like autocorrelation structure, and we are also concerned with a substantial extent of cross-correlation. However, it can be seen that for both sub-series, the counts under sales promotion are at a higher level than without sales promotion. This indicates that the sales promotion is helpful to stimulate the number of sold items, and might thus be a relevant circumstance state. If computing the state-dependent sample means and variances (as required for YW estimation anyway, recall Section 3.1), one gets the values of Table 1. Comparing the means across the states, the visual impression from Figure 1 is confirmed, which is that counts are larger (in the mean) in state 1 (promotion) than in state 2. But, it is also interesting to compare the corresponding means and variances. Keeping in mind that the sub-series are rather short, such that variations are natural, the overall impression is that means and variances are reasonably close to each other, i.e., a model with state-dependent equidispersion could be suitable for the data. Together with the aforementioned substantial extent of cross-correlation, it is thus reasonable to try the novel CuBINAR(1) model for the sales counts data.

To evaluate the performance of our new model, we fit the CuBINAR(1) model to the data, and as competitors, we consider the classical (stationary) Poi-BINAR(1) model (2) of [11] on the one hand, and the RE-BINAR(1) model of [25] (recall Remark 3) on the other hand. Model fitting is performed via the CML approach, where the numerical optimization is initialized by the YW estimates (recall Section 3 and Section 4). The estimation results are summarized in Table 2. We also computed approximate standard errors as described in Section 3.2, but since the time series is rather short, the dependence parameters ${\hat{\alpha }}_{1},{\hat{\alpha }}_{2},\hat{\phi }$ are not significant on a 5%-level. As the estimates ${\hat{\lambda }}_{i}\left(s_{t}\right)$ in the CuBINAR(1) model refer to the marginal mean of $X_{i,t}\left(s_{t}\right)$, we convert the means of the BINAR(1)’s innovation terms $\varepsilon_{i,t}$ into the marginal mean of $X_{i,t}$ in order to make the results comparable. That is, the estimates ${\hat{\lambda }}_{1}$ and ${\hat{\lambda }}_{2}$ of the BINAR(1) model represent the marginal means of $X_{i,t}$. We can see that the CuBINAR(1)’s estimates ${\hat{\lambda }}_{i}\left(1\right)$, i=1,2, of both sub-series under state 1 are larger than those under state 2, which confirms that the sales promotion increases the number of sold items. The corresponding ${\hat{\lambda }}_{i}$ of the BINAR(1) model are located between ${\hat{\lambda }}_{i}\left(1\right)$ and ${\hat{\lambda }}_{i}\left(2\right)$, whereas the RE-BINAR(1)’s estimates differ quite a lot in some cases (and also deviate from the sample means in Table 1). It is also interesting to note that the values of ${\hat{\alpha }}_{1},{\hat{\alpha }}_{2},\hat{\phi }$ are smaller for CuBINAR(1) than for BINAR(1), which is reasonable as part of the CuBINAR(1)’s dependence is explained by the circumstance states. Furthermore, the estimate $\hat{\phi }$ is clearly larger than zero, i.e., the ability of the CuBINAR(1) model to incorporate additional cross-dependence (recall Remark 3) turns out to be beneficial in view of the substantial extent of cross-correlation observed in Figure 1.

To assess the performance of the fitted models, we first compare the root mean square errors (RMSEs) between the observations and predicted values. More precisely, the RMSE values are the square-roots of sums of the form $\sum_{t}{\left(x_{i,t}-E\left[X_{i,t}\left(s_{t}\right)\;|\;x_{i,t-1}\left(s_{t-1}\right)\right]\right)}^{2}$, divided by the number of summands. Here, we distinguish two cases. The in-sample RMSE is computed by using the model fits of Table 2 and by summing for t=2,…,n. For the out-of-sample RMSEs, we omitted the last 10 observations during model fitting, and then the sum was taken about t=n−9,…,n. Obviously, the RMSE performances of the CuBINAR(1) model are better than those of both the RE-BINAR(1) and BINAR(1) model.

In addition to the RMSE, we also adopt scoring rules and Akaike’s information criterion (AIC) for model choice. Regarding the scoring rules, we use the logarithmic score defined as

$$\begin{array}{c} S_{ls}\left(p_{\cdot |x_{t-1}},x_{t}\right):=-\ln p_{x_{t}|x_{t-1}}. \end{array}$$

The mean score $\frac{1}{n-1}\sum_{t=2}^{n}S_{ls}\left(p_{\cdot |x_{t-1}},x_{t}\right)$ is used to assess the overall performance of the model. Smaller score values indicate that the predictive distribution provided by the fitted model is in better agreement with the true predictive distribution, which implies a better fit of the model. Analogously, smaller values of the AIC indicate a better model. From Table 3, we recognize that both the AIC and the logarithmic score of the CuBINAR(1) model are smaller than those of the competing models. Altogether, the CuBINAR(1) model clearly outperforms both competitors. Regarding the BINAR(1) model, our newly proposed model can better fit the sales count data by utilizing the dependence to the underlying circumstance. The superior performance compared to the RE-BINAR(1) model can be explained from two types of sample properties noted in the beginning of this section. First, the data exhibit notable cross-correlation, but only the CuBINAR(1) model has an additional cross-dependence parameter. Second, conditioned on the different states, the sales counts are close to equidispersion, which is accounted for by the CuBINAR(1)’s Poisson distributions. The RE-BINAR(1) model with its geometric distributions, by contrast, is designed for strongly overdispersed data, but which does not apply to the sales counts data.

While CuBINAR(1) model performs best among the candidate models, it remains to assess its overall model adequacy. First, we analyzed the corresponding standardized Pearson residuals defined by

$$\frac{x_{i,t}-E\left[X_{i,t}\left(s_{t}\right)\;|\;x_{i,t-1}\left(s_{t-1}\right)\right]}{\sqrt{Var(X_{i,t}\left(s_{t}\right)\;|\;x_{i,t-1}\left(s_{t-1}\right))}}\;\mathrm{for}\;i=1,2\;\mathrm{and}\;t=2,3,\ldots$$

**Figure 2:**
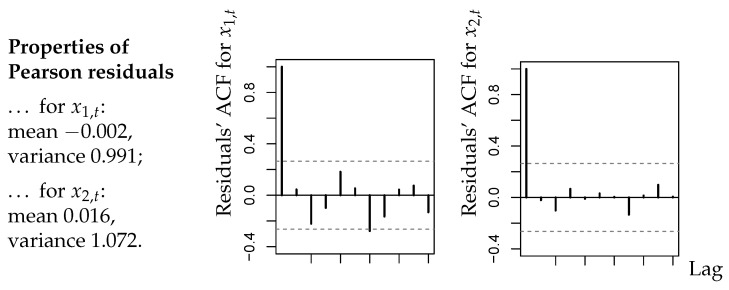
Bivariate sales counts from Section 5: sample means, variances, and ACFs of Pearson residuals with respect to fitted CuBINAR(1) model.

**Figure 3:**
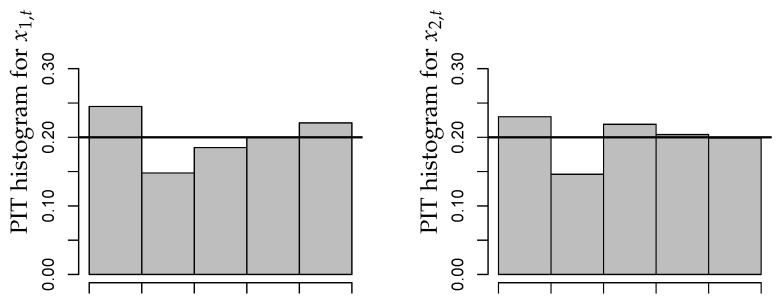
Bivariate sales counts from Section 5: PIT histograms with respect to fitted CuBINAR(1) model.

A summary of results is provided by Figure 2. As explained in Section 2.4 of [1], the residuals of an adequate model should have a mean close to zero, a variance close to one, and they should not be autocorrelated. From Figure 2, we conclude that these criteria are satisfied in good approximation. It is also worth noting that there exist no significant cross-correlations between the residuals series. We also computed the PIT histograms for the fitted CuBINAR(1) model, as these are another common approach for checking the model adequacy (see Section 2.4 in [1]). But, since the sample size is rather short, the PIT histograms in Figure 3 look a bit “spiky”. Nevertheless, they exhibit no systematic deviation from uniformity, such as a (inverse) U-shape. Therefore, they do not contradict to the fitted CuBINAR(1) model. Altogether, our novel CuBINAR(1) model appears to adequately describe the bivariate sales counts data.

## 6. Conclusions

In this paper, we proposed the new circumstance-driven bivariate INAR(1) model, which can be applied to bivariate count time series that have different marginal means caused by an underlying circumstance factor. Important stochastic properties of the new model were discussed. We applied and analyzed the Yule–Walker and conditional maximum likelihood method to estimate the unknown parameter values. The consistency of the estimators was also confirmed by our simulation study where the estimation results converge quickly to the true parameter values with increasing sample size. For the presented real-data application on sales counts, our new model outperforms the ordinary BINAR(1) model. As a possible direction for future research, we suggest equipping models for multivariate count time series with a self-exciting threshold mechanism, similar to the recent work by [27]. Another important topic would be the case where the states cannot be observed (latent states, in analogy to the Hidden-Markov model [26]). Then, the CuBINAR(1)’s model definition and estimation approaches need to be adapted, which should be performed in future research.

## Figures and Tables

**Table 1:** State-dependent sample means and variances of sales counts data.

	State 1		State 2	
	**Mean**	**Var**	**Mean**	**Var**
$x_{1,t}$	3.111	3.046	2.132	2.063
$x_{2,t}$	4.500	3.206	1.553	1.876

**Table 2:** CML parameter estimates of sales counts data.

Model	CuBINAR(1)		RE-BINAR(1)		BINAR(1)	
	${\hat{\alpha }}_{1}$	0.265	${\hat{\alpha }}_{1}$	0.609	${\hat{\alpha }}_{1}$	0.288
	${\hat{\alpha }}_{2}$	0.215	${\hat{\alpha }}_{2}$	0.619	${\hat{\alpha }}_{2}$	0.396
	${\hat{\lambda }}_{1}\left(1\right)$	2.929	${\hat{\lambda }}_{1}\left(1\right)$	1.823	${\hat{\lambda }}_{1}$	2.480
	${\hat{\lambda }}_{1}\left(2\right)$	2.266	${\hat{\lambda }}_{1}\left(2\right)$	2.090		
	${\hat{\lambda }}_{2}\left(1\right)$	4.187	${\hat{\lambda }}_{2}\left(1\right)$	3.589	${\hat{\lambda }}_{2}$	2.467
	${\hat{\lambda }}_{2}\left(2\right)$	1.586	${\hat{\lambda }}_{2}\left(2\right)$	1.322		
	$\hat{\phi }$	0.250			$\hat{\phi }$	0.312

**Table 3:** AIC, logarithmic score, and RMSE of sales counts data.

Model:	AIC	Logarithmic Score	in-RMSE	out-RMSE
CuBINAR(1)	399.8	3.507	$x_{1,t}$: 1.468	1.849
			$x_{2,t}$: 1.456	1.864
RE−BINAR(1)	427.8	3.780	$x_{1,t}$: 1.607	2.163
			$x_{2,t}$: 1.566	2.139
BINAR(1)	418.1	3.710	$x_{1,t}$: 1.504	1.887
			$x_{2,t}$: 1.761	2.408

## Data Availability

Publicly available datasets were analyzed in this study. These data can be found here: https://www.chicagobooth.edu/research/kilts/datasets/dominicks, accessed on 10 November 2021.

## Appendix A. Tabulated Simulation Results for Section 4

**Table A1:** Parameter estimation under three states; see Section 4.

Transition Matrix 1: $\left[\begin{array}{ccc} 0.4 & 0.3 & 0.3\\ 0.3 & 0.4 & 0.3\\ 0.3 & 0.3 & 0.4 \end{array}\right]$									
$(\alpha_{1},\alpha_{2},\phi ,\phi^{*},\lambda_{1}\left(1\right),\lambda_{1}\left(2\right),\lambda_{1}\left(3\right),\lambda_{2}\left(1\right),\lambda_{2}\left(2\right),\lambda_{3}\left(3\right))=\left(0.15,0.2,0.5,0.485,1,2,3,4,5,6\right)$									
Yule–Walker									
*n*	$\alpha_{1}$	$\alpha_{2}$	ϕ	$\lambda_{1}\left(1\right)$	$\lambda_{1}\left(2\right)$	$\lambda_{1}\left(3\right)$	$\lambda_{2}\left(1\right)$	$\lambda_{2}\left(2\right)$	$\lambda_{2}\left(3\right)$
300	0.1448	0.1913	0.4956	1.0016	1.9983	3.0000	3.9994	5.0004	6.0016
sd	0.0647	0.0636	0.1970	0.1070	0.1515	0.1855	0.2185	0.2475	0.2705
bias	−0.0052	−0.0087	−0.0044	0.0016	−0.0017	0.0000	−0.0006	0.0004	0.0016
900	0.1475	0.1971	0.4991	0.9998	1.9979	3.0005	3.9992	4.9988	6.0012
sd	0.0395	0.0370	0.1163	0.0613	0.0868	0.1069	0.1283	0.1425	0.1548
bias	−0.0025	−0.0029	−0.0009	−0.0002	−0.0021	0.0005	−0.0008	−0.0012	0.0012
1500	0.1481	0.1987	0.4989	1.0001	2.0012	3.0014	3.9993	5.0009	6.0023
sd	0.0308	0.0292	0.0893	0.0475	0.0680	0.0827	0.0970	0.1094	0.1192
bias	−0.0019	−0.0013	−0.0011	0.0001	0.0012	0.0014	−0.0007	0.0009	0.0023
2100	0.1487	0.1988	0.4993	0.9998	1.9994	3.0003	4.0001	5.0010	6.0009
sd	0.0258	0.0247	0.0763	0.0402	0.0567	0.0702	0.0822	0.0919	0.1004
bias	−0.0013	−0.0012	−0.0007	−0.0002	−0.0006	0.0003	0.0001	0.0010	0.0009
Conditional Maximum Likelihood									
*n*	$\alpha_{1}$	$\alpha_{2}$	ϕ	$\lambda_{1}\left(1\right)$	$\lambda_{1}\left(2\right)$	$\lambda_{1}\left(3\right)$	$\lambda_{2}\left(1\right)$	$\lambda_{2}\left(2\right)$	$\lambda_{2}\left(3\right)$
300	0.1519	0.1984	0.4949	0.9997	1.9994	3.0017	3.9986	5.0008	6.0011
sd	0.0519	0.0548	0.1508	0.1042	0.1490	0.1834	0.2133	0.2414	0.2652
bias	0.0019	−0.0016	0.0099	−0.0003	−0.0006	0.0017	−0.0014	0.0008	0.0011
900	0.1502	0.1991	0.4886	0.9994	1.9978	3.0012	3.9994	4.9984	6.0014
sd	0.0294	0.0306	0.0833	0.0590	0.0850	0.1045	0.1249	0.1389	0.1515
bias	0.0002	−0.0009	0.0036	−0.0006	−0.0022	0.0012	−0.0006	−0.0016	0.0014
1500	0.1499	0.2001	0.4872	0.9996	2.0016	3.0015	3.9991	5.0012	6.0023
sd	0.0225	0.0241	0.0640	0.0454	0.0663	0.0813	0.0942	0.1067	0.1163
bias	−0.0001	0.0001	0.0022	−0.0004	0.0016	0.0015	−0.0009	0.0012	0.0023
2100	0.1500	0.1998	0.4863	0.9995	1.9996	3.0006	4.0000	5.0009	6.0012
sd	0.0187	0.0201	0.0541	0.0388	0.0555	0.0686	0.0800	0.0896	0.0974
bias	0.0000	−0.0002	0.0013	−0.0005	−0.0004	0.0006	0.0000	0.0009	0.0012

**Table A2:** Parameter estimation under three states; see Section 4.

Transition Matrix 1: $\left[\begin{array}{ccc} 0.4 & 0.3 & 0.3\\ 0.3 & 0.4 & 0.3\\ 0.3 & 0.3 & 0.4 \end{array}\right]$									
$(\alpha_{1},\alpha_{2},\phi ,\phi^{*},\lambda_{1}\left(1\right),\lambda_{1}\left(2\right),\lambda_{1}\left(3\right),\lambda_{2}\left(1\right),\lambda_{2}\left(2\right),\lambda_{3}\left(3\right))=\left(0.15,0.5,0.5,0.4625,1,2,3,4,5,6\right)$									
Yule–Walker									
*n*	$\alpha_{1}$	$\alpha_{2}$	ϕ	$\lambda_{1}\left(1\right)$	$\lambda_{1}\left(2\right)$	$\lambda_{1}\left(3\right)$	$\lambda_{2}\left(1\right)$	$\lambda_{2}\left(2\right)$	$\lambda_{2}\left(3\right)$
300	0.1440	0.4828	0.4949	0.9996	2.0003	3.0016	3.9977	4.9944	6.0038
sd	0.0642	0.0925	0.2013	0.1059	0.1506	0.1848	0.2618	0.2971	0.3273
bias	−0.0060	−0.0172	−0.0051	−0.0004	0.0003	0.0016	−0.0023	−0.0056	0.0038
900	0.1474	0.4947	0.4981	0.9993	2.0017	2.9991	3.9978	5.0001	5.9993
sd	0.0396	0.0540	0.1209	0.0612	0.0872	0.1064	0.1516	0.1702	0.1882
bias	−0.0026	−0.0053	−0.0019	−0.0007	0.0017	−0.0009	−0.0022	0.0001	−0.0007
1500	0.1484	0.4956	0.4978	0.9996	2.0006	3.0014	3.9993	5.0014	6.0018
sd	0.0300	0.0413	0.0944	0.0477	0.0676	0.0833	0.1171	0.1320	0.1435
bias	−0.0016	−0.0044	−0.0022	−0.0004	0.0006	0.0014	−0.0007	0.0014	0.0018
2100	0.1490	0.4974	0.4997	0.9998	1.9991	2.9992	4.0012	5.0011	6.0006
sd	0.0255	0.0355	0.0797	0.0407	0.0569	0.0702	0.1009	0.1114	0.1226
bias	−0.0010	−0.0026	−0.0003	−0.0002	−0.0009	−0.0008	0.0012	0.0011	0.0006
Conditional Maximum Likelihood									
*n*	$\alpha_{1}$	$\alpha_{2}$	$\phi^{*}$	$\lambda_{1}\left(1\right)$	$\lambda_{1}\left(2\right)$	$\lambda_{1}\left(3\right)$	$\lambda_{2}\left(1\right)$	$\lambda_{2}\left(2\right)$	$\lambda_{2}\left(3\right)$
300	0.1511	0.5004	0.4704	0.9973	2.0017	3.0034	3.9971	4.9980	6.0027
sd	0.0512	0.0387	0.1315	0.1031	0.1484	0.1820	0.2360	0.2685	0.2935
bias	0.0011	0.0004	0.0079	−0.0027	0.0017	0.0034	−0.0030	−0.0020	0.0027
900	0.1501	0.5004	0.4656	0.9988	2.0017	2.9999	3.9987	5.0002	5.9983
sd	0.0289	0.0212	0.0729	0.0588	0.0853	0.1041	0.1355	0.1542	0.1683
bias	0.0001	0.0004	0.0031	−0.0012	0.0017	−0.0001	−0.0013	0.0002	−0.0017
1500	0.1502	0.4999	0.4637	1.0002	1.9998	3.0003	3.9997	4.9997	5.9996
sd	0.0219	0.0166	0.0562	0.0462	0.0661	0.0808	0.1047	0.1182	0.1296
bias	0.0002	−0.0001	0.0012	0.0002	−0.0002	0.0003	−0.0003	−0.0003	−0.0004
2100	0.1495	0.5000	0.4630	0.9992	1.9987	3.0010	4.0000	4.9998	6.0004
sd	0.0192	0.0141	0.0477	0.0399	0.0576	0.0712	0.0921	0.1056	0.1133
bias	−0.0005	0.0000	0.0005	−0.0008	−0.0013	0.0009	0.0000	−0.0002	0.0004

**Table A3:** Parameter estimation under three states; see Section 4.

Transition Matrix 1: $\left[\begin{array}{ccc} 0.4 & 0.3 & 0.3\\ 0.3 & 0.4 & 0.3\\ 0.3 & 0.3 & 0.4 \end{array}\right]$									
$(\alpha_{1},\alpha_{2},\phi ,\phi^{*},\lambda_{1}\left(1\right),\lambda_{1}\left(2\right),\lambda_{1}\left(3\right),\lambda_{2}\left(1\right),\lambda_{2}\left(2\right),\lambda_{3}\left(3\right))=\left(0.4,0.25,1,0.9,3,4,5,2,3,4\right)$									
Yule–Walker									
*n*	$\alpha_{1}$	$\alpha_{2}$	ϕ	$\lambda_{1}\left(1\right)$	$\lambda_{1}\left(2\right)$	$\lambda_{1}\left(3\right)$	$\lambda_{2}\left(1\right)$	$\lambda_{2}\left(2\right)$	$\lambda_{2}\left(3\right)$
300	0.3846	0.2392	0.9848	3.0039	4.0036	5.0040	1.9994	2.9987	3.9987
sd	0.0801	0.0681	0.2421	0.2121	0.2471	0.2768	0.1582	0.1954	0.2278
bias	−0.0154	−0.0108	−0.0152	0.0039	0.0036	0.0040	−0.0006	−0.0013	−0.0013
900	0.3947	0.2465	0.9958	3.0015	4.0002	5.0016	2.0005	2.9995	4.0022
sd	0.0469	0.0403	0.1393	0.1225	0.1414	0.1560	0.0921	0.1127	0.1296
bias	−0.0053	−0.0035	−0.0042	0.0015	0.0002	0.0016	0.0005	−0.0005	0.0022
1500	0.3971	0.2485	0.9957	3.0002	4.0008	5.0009	1.9997	2.9999	3.9994
sd	0.0367	0.0311	0.1087	0.0952	0.1095	0.1221	0.0711	0.0859	0.1009
bias	−0.0029	−0.0015	−0.0043	0.0002	0.0008	0.0009	−0.0003	−0.0001	−0.0006
2100	0.3980	0.2486	0.9972	3.0013	3.9994	4.9992	2.0009	2.9996	3.9993
sd	0.0310	0.0263	0.0925	0.0798	0.0924	0.1024	0.0604	0.0737	0.0838
bias	−0.0020	−0.0014	−0.0028	0.0013	−0.0006	−0.0008	0.0009	−0.0004	−0.0007
Conditional Maximum Likelihood									
*n*	$\alpha_{1}$	$\alpha_{2}$	$\phi^{*}$	$\lambda_{1}\left(1\right)$	$\lambda_{1}\left(2\right)$	$\lambda_{1}\left(3\right)$	$\lambda_{2}\left(1\right)$	$\lambda_{2}\left(2\right)$	$\lambda_{2}\left(3\right)$
300	0.4034	0.2502	0.9102	2.9864	4.0013	5.0111	1.9898	2.9997	4.0060
sd	0.0445	0.0512	0.1490	0.2026	0.2416	0.2645	0.1567	0.1927	0.2260
bias	0.0034	0.0002	0.0102	−0.0136	0.0013	0.0111	−0.0102	−0.0003	0.0060
900	0.4015	0.2498	0.9056	2.9958	4.0011	5.0059	1.9964	2.9999	4.0020
sd	0.0247	0.0276	0.0816	0.1160	0.1372	0.1520	0.0886	0.1114	0.1295
bias	0.0015	−0.0002	0.0056	−0.0042	0.0011	0.0059	−0.0036	−0.0001	0.0020
1500	0.4007	0.2504	0.9036	2.9991	4.0010	5.0058	2.0000	2.9995	4.0015
sd	0.0190	0.0213	0.0641	0.0885	0.1053	0.1182	0.0676	0.0865	0.0995
bias	0.0007	0.0004	0.0036	−0.0009	0.0010	0.0058	0.0000	−0.0005	0.0015
2100	0.4005	0.2499	0.9039	2.9980	4.0000	5.0021	1.9990	2.9998	4.0023
sd	0.0157	0.0183	0.0540	0.0744	0.0898	0.0992	0.0572	0.0731	0.0841
bias	0.0005	−0.0001	0.0039	−0.0020	0.0000	0.0021	−0.0010	−0.0002	0.0023

**Table A4:** Parameter estimation under three states; see Section 4.

Transition Matrix 2: $\left[\begin{array}{ccc} 0.6 & 0.3 & 0.1\\ 0.1 & 0.6 & 0.3\\ 0.3 & 0.1 & 0.6 \end{array}\right]$									
$(\alpha_{1},\alpha_{2},\phi ,\phi^{*},\lambda_{1}\left(1\right),\lambda_{1}\left(2\right),\lambda_{1}\left(3\right),\lambda_{2}\left(1\right),\lambda_{2}\left(2\right),\lambda_{3}\left(3\right))=\left(0.15,0.2,0.5,0.485,1,2,3,4,5,6\right)$									
Yule–Walker									
*n*	$\alpha_{1}$	$\alpha_{2}$	ϕ	$\lambda_{1}\left(1\right)$	$\lambda_{1}\left(2\right)$	$\lambda_{1}\left(3\right)$	$\lambda_{2}\left(1\right)$	$\lambda_{2}\left(2\right)$	$\lambda_{2}\left(3\right)$
300	0.1419	0.1894	0.4942	1.0001	2.0012	2.9994	3.9989	5.0001	5.9982
sd	0.0630	0.0648	0.1956	0.1107	0.1547	0.1896	0.2290	0.2547	0.2769
bias	−0.0081	−0.0106	−0.0058	0.0001	0.0012	−0.0006	−0.0011	0.0001	−0.0018
900	0.1471	0.1969	0.4997	1.0000	2.0008	2.9998	3.9990	5.0009	5.9996
sd	0.0380	0.0372	0.1146	0.0636	0.0886	0.1092	0.1299	0.1450	0.1605
bias	−0.0029	−0.0031	−0.0003	0.0000	0.0008	−0.0002	−0.0010	0.0009	−0.0004
1500	0.1482	0.1982	0.4972	1.0000	2.0015	3.0009	4.0010	4.9999	6.0002
sd	0.0294	0.0287	0.0894	0.0492	0.0689	0.0851	0.1011	0.1146	0.1254
bias	−0.0018	−0.0018	−0.0028	0.0000	0.0015	0.0009	0.0010	−0.0001	0.0002
2100	0.1484	0.1985	0.5000	0.9997	2.0005	3.0010	4.0002	4.9992	6.0008
sd	0.0250	0.0246	0.0761	0.0413	0.0593	0.0725	0.0852	0.0955	0.1036
bias	−0.0016	−0.0015	0.0000	−0.0003	0.0005	0.0010	0.0002	−0.0008	0.0008
Conditional Maximum Likelihood									
*n*	$\alpha_{1}$	$\alpha_{2}$	$\phi^{*}$	$\lambda_{1}\left(1\right)$	$\lambda_{1}\left(2\right)$	$\lambda_{1}\left(3\right)$	$\lambda_{2}\left(1\right)$	$\lambda_{2}\left(2\right)$	$\lambda_{2}\left(3\right)$
300	0.1504	0.1961	0.4953	0.9971	2.0027	3.0024	3.9974	5.0006	5.9996
sd	0.0537	0.0559	0.1514	0.1081	0.1528	0.1867	0.2234	0.2499	0.2706
bias	0.0004	−0.0039	0.0103	−0.0029	0.0027	0.0024	−0.0026	0.0006	−0.0004
900	0.1492	0.1985	0.4871	0.9992	2.0008	3.0013	3.9995	4.9984	6.0016
sd	0.0295	0.0317	0.0833	0.0614	0.0882	0.1094	0.1296	0.1412	0.1553
bias	−0.0008	−0.0015	0.0021	−0.0008	0.0008	0.0013	−0.0005	−0.0016	0.0016
1500	0.1502	0.1997	0.4873	0.9996	1.9993	3.0005	4.0004	5.0002	6.0016
sd	0.0230	0.0241	0.0647	0.0474	0.0687	0.0846	0.0996	0.1111	0.1211
bias	0.0002	−0.0003	0.0023	−0.0004	−0.0007	0.0005	0.0004	0.0002	0.0016
2100	0.1496	0.1996	0.4865	0.9997	2.0006	3.0009	4.0004	5.0002	5.9995
sd	0.0192	0.0203	0.0540	0.0402	0.0574	0.0704	0.0834	0.0936	0.1017
bias	−0.0004	−0.0004	0.0015	−0.0003	0.0006	0.0009	0.0004	0.0002	−0.0005

**Table A5:** Parameter estimation under three states; see Section 4.

Transition Matrix 2: $\left[\begin{array}{ccc} 0.6 & 0.3 & 0.1\\ 0.1 & 0.6 & 0.3\\ 0.3 & 0.1 & 0.6 \end{array}\right]$									
$(\alpha_{1},\alpha_{2},\phi ,\phi^{*},\lambda_{1}\left(1\right),\lambda_{1}\left(2\right),\lambda_{1}\left(3\right),\lambda_{2}\left(1\right),\lambda_{2}\left(2\right),\lambda_{3}\left(3\right))=\left(0.15,0.5,0.5,0.4625,1,2,3,4,5,6\right)$									
Yule–Walker									
*n*	$\alpha_{1}$	$\alpha_{2}$	ϕ	$\lambda_{1}\left(1\right)$	$\lambda_{1}\left(2\right)$	$\lambda_{1}\left(3\right)$	$\lambda_{2}\left(1\right)$	$\lambda_{2}\left(2\right)$	$\lambda_{2}\left(3\right)$
300	0.1426	0.4786	0.4932	0.9983	1.9989	2.9982	3.9964	5.0004	6.0066
sd	0.0636	0.0915	0.2039	0.1093	0.1592	0.1907	0.2781	0.3175	0.3457
bias	−0.0074	−0.0214	−0.0068	−0.0017	−0.0011	−0.0018	−0.0036	0.0004	0.0066
900	0.1466	0.4932	0.4956	0.9995	1.9995	3.0010	3.9997	4.9992	5.9999
sd	0.0382	0.0539	0.1198	0.0638	0.0893	0.1108	0.1602	0.1820	0.1986
bias	−0.0034	−0.0068	−0.0044	−0.0005	−0.0005	0.0010	−0.0003	−0.0008	−0.0001
1500	0.1482	0.4954	0.4979	1.0002	2.0005	3.0012	4.0001	5.0005	5.9997
sd	0.0296	0.0421	0.0938	0.0489	0.0693	0.0839	0.1260	0.1415	0.1535
bias	−0.0018	−0.0046	−0.0021	0.0002	0.0005	0.0012	0.0001	0.0005	−0.0003
2100	0.1487	0.4971	0.4979	0.9999	2.0000	2.9988	4.0007	4.9986	6.0005
sd	0.0248	0.0353	0.0788	0.0420	0.0593	0.0718	0.1059	0.1196	0.1290
bias	−0.0013	−0.0029	−0.0021	−0.0001	0.0000	−0.0012	0.0007	−0.0014	0.0005
Conditional Maximum Likelihood									
*n*	$\alpha_{1}$	$\alpha_{2}$	$\phi^{*}$	$\lambda_{1}\left(1\right)$	$\lambda_{1}\left(2\right)$	$\lambda_{1}\left(3\right)$	$\lambda_{2}\left(1\right)$	$\lambda_{2}\left(2\right)$	$\lambda_{2}\left(3\right)$
300	0.1499	0.5000	0.4711	0.9975	2.0019	3.0042	3.9947	5.0020	6.0053
sd	0.0535	0.0396	0.1323	0.1069	0.1523	0.1898	0.2477	0.2805	0.3040
bias	−0.0001	0.0000	0.0086	−0.0025	0.0019	0.0042	−0.0053	0.0020	0.0053
900	0.1497	0.5003	0.4642	0.9989	2.0002	2.9998	3.9987	5.0015	6.0001
sd	0.0297	0.0219	0.0742	0.0606	0.0879	0.1092	0.1402	0.1614	0.1723
bias	−0.0003	0.0003	0.0017	−0.0011	0.0002	−0.0002	−0.0013	0.0015	0.0001
1500	0.1498	0.4999	0.4632	0.9995	2.0004	3.0014	4.0002	5.0007	6.0029
sd	0.0227	0.0168	0.0568	0.0474	0.0672	0.0828	0.1104	0.1233	0.1349
bias	−0.0002	−0.0001	0.0007	−0.0005	0.0004	0.0014	0.0002	0.0007	0.0029
2100	0.1496	0.5000	0.4640	0.9998	1.9994	2.9990	4.0000	5.0005	6.0002
sd	0.0192	0.0142	0.0483	0.0394	0.0575	0.0705	0.0939	0.1070	0.1155
bias	−0.0004	0.0000	0.0015	−0.0002	−0.0006	−0.0010	0.0000	0.0005	0.0002

**Table A6:** Parameter estimation under three states; see Section 4.

Transition Matrix 2: $\left[\begin{array}{ccc} 0.6 & 0.3 & 0.1\\ 0.1 & 0.6 & 0.3\\ 0.3 & 0.1 & 0.6 \end{array}\right]$									
$(\alpha_{1},\alpha_{2},\phi ,\phi^{*},\lambda_{1}\left(1\right),\lambda_{1}\left(2\right),\lambda_{1}\left(3\right),\lambda_{2}\left(1\right),\lambda_{2}\left(2\right),\lambda_{3}\left(3\right))=\left(0.4,0.25,1,0.9,3,4,5,2,3,4\right)$									
Yule–Walker									
*n*	$\alpha_{1}$	$\alpha_{2}$	ϕ	$\lambda_{1}\left(1\right)$	$\lambda_{1}\left(2\right)$	$\lambda_{1}\left(3\right)$	$\lambda_{2}\left(1\right)$	$\lambda_{2}\left(2\right)$	$\lambda_{2}\left(3\right)$
300	0.3840	0.2366	0.9830	3.0022	4.0036	5.0012	1.9986	3.0006	4.0033
sd	0.0801	0.0675	0.2392	0.2246	0.2611	0.2950	0.1645	0.2040	0.2350
bias	−0.0160	−0.0134	−0.0170	0.0022	0.0036	0.0012	−0.0014	0.0006	0.0033
900	0.3948	0.2460	0.9948	2.9996	4.0004	5.0020	2.0000	2.9990	4.0024
sd	0.0466	0.0405	0.1397	0.1299	0.1524	0.1681	0.0963	0.1176	0.1351
bias	−0.0052	−0.0040	−0.0052	−0.0004	0.0004	0.0020	0.0000	−0.0010	0.0024
1500	0.3973	0.2473	0.9973	3.0008	4.0004	5.0004	1.9991	3.0007	4.0008
sd	0.0366	0.0308	0.1086	0.1004	0.1153	0.1290	0.0736	0.0897	0.1041
bias	−0.0027	−0.0027	−0.0027	0.0008	0.0004	0.0004	−0.0009	0.0007	0.0008
2100	0.3973	0.2480	0.9968	3.0003	4.0012	5.0002	1.9994	3.0009	3.9995
sd	0.0309	0.0262	0.0915	0.0846	0.0981	0.1100	0.0623	0.0766	0.0887
bias	−0.0027	−0.0020	−0.0032	0.0003	0.0012	0.0002	−0.0006	0.0009	−0.0005
Conditional Maximum Likelihood									
*n*	$\alpha_{1}$	$\alpha_{2}$	$\phi^{*}$	$\lambda_{1}\left(1\right)$	$\lambda_{1}\left(2\right)$	$\lambda_{1}\left(3\right)$	$\lambda_{2}\left(1\right)$	$\lambda_{2}\left(2\right)$	$\lambda_{2}\left(3\right)$
300	0.4034	0.2502	0.9102	2.9864	4.0013	5.0111	1.9898	2.9997	4.0060
sd	0.0445	0.0512	0.1490	0.2026	0.2416	0.2645	0.1567	0.1927	0.2260
bias	0.0034	0.0002	0.0102	−0.0136	0.0013	0.0111	−0.0102	−0.0003	0.0060
900	0.4015	0.2495	0.9044	2.9950	4.0005	5.0060	1.9969	2.9996	4.0018
sd	0.0248	0.0278	0.0823	0.1159	0.1373	0.1520	0.0883	0.1105	0.1273
bias	0.0015	−0.0005	0.0044	−0.0050	0.0005	0.0060	−0.0031	−0.0004	0.0018
1500	0.4010	0.2495	0.9024	2.9976	4.0011	5.0044	1.9972	3.0006	4.0021
sd	0.0186	0.0213	0.0634	0.0894	0.1054	0.1168	0.0683	0.0867	0.1000
bias	0.0010	−0.0005	0.0024	−0.0024	0.0011	0.0044	−0.0029	0.0006	0.0021
2100	0.4006	0.2502	0.9025	2.9976	3.9993	5.0023	1.9980	2.9992	3.9995
sd	0.0159	0.0181	0.0544	0.0741	0.0899	0.0983	0.0566	0.0733	0.0835
bias	0.0006	0.0002	0.0025	−0.0024	−0.0007	0.0023	−0.0020	−0.0008	−0.0005

**Table A7:** Parameter estimation under two states; see Section 4.

Transition Matrix 1: $\left[\begin{array}{cc} 0.4 & 0.6\\ 0.6 & 0.4 \end{array}\right]$							
$(\alpha_{1},\alpha_{2},\phi ,\phi^{*},\lambda_{1}\left(1\right),\lambda_{1}\left(2\right),\lambda_{2}\left(1\right),\lambda_{2}\left(2\right))=\left(0.15,0.5,0.5,0.4625,1,3,3,5\right)$							
Yule–Walker							
	$\alpha_{1}$	$\alpha_{2}$	ϕ	$\lambda_{1}\left(1\right)$	$\lambda_{1}\left(2\right)$	$\lambda_{2}\left(1\right)$	$\lambda_{2}\left(2\right)$
300	0.1487	0.4889	0.4997	0.9988	2.9996	2.9959	5.0047
sd	0.0684	0.0938	0.1877	0.0863	0.1513	0.1962	0.2518
bias	−0.0013	−0.0111	−0.0002	−0.0012	−0.0004	−0.0041	0.0047
900	0.1483	0.4960	0.4989	1.0000	3.0014	2.9978	4.9989
sd	0.0418	0.0545	0.1126	0.0502	0.0872	0.1139	0.1461
bias	−0.0017	−0.0040	−0.0010	0.0000	0.0014	−0.0022	−0.0011
1500	0.1490	0.4980	0.4994	1.0001	3.0019	2.9993	5.0025
sd	0.0327	0.0429	0.0863	0.0390	0.0669	0.0878	0.1126
bias	−0.0010	−0.0020	−0.0005	0.0001	0.0019	−0.0007	0.0025
2100	0.1491	0.4983	0.4997	0.9998	2.9999	2.9994	5.0009
sd	0.0276	0.0361	0.0739	0.0326	0.0576	0.0737	0.0949
bias	−0.0009	−0.0017	−0.0002	−0.0002	−0.0001	−0.0006	0.0009
Conditional Maximum Likelihood							
	$\alpha_{1}$	$\alpha_{2}$	$\phi^{*}$	$\lambda_{1}\left(1\right)$	$\lambda_{1}\left(2\right)$	$\lambda_{2}\left(1\right)$	$\lambda_{2}\left(2\right)$
300	0.1503	0.5159	0.4802	0.9955	3.0026	2.9878	5.0140
sd	0.0460	0.0368	0.0867	0.0844	0.1498	0.1850	0.2362
bias	0.0003	0.0159	0.0177	−0.0045	0.0026	−0.0122	0.0140
900	0.1491	0.5112	0.4704	0.9987	2.9999	2.9922	5.0088
sd	0.0246	0.0204	0.0486	0.0493	0.0850	0.1065	0.1370
bias	−0.0009	0.0112	0.0079	−0.0013	−0.0001	−0.0078	0.0088
1500	0.1481	0.5091	0.4676	0.9977	3.0012	2.9941	5.0087
sd	0.0185	0.0164	0.0365	0.0383	0.0647	0.0821	0.1058
bias	−0.0019	0.0091	0.0051	−0.0023	0.0012	−0.0059	0.0087
2100	0.1485	0.5078	0.4658	0.9987	3.0004	2.9918	5.0041
sd	0.0155	0.0139	0.0290	0.0317	0.0578	0.0703	0.0909
bias	−0.0015	0.0078	0.0033	−0.0013	0.0004	−0.0082	0.0041

**Table A8:** Parameter estimation under two states; see Section 4.

Transition Matrix 1: $\left[\begin{array}{cc} 0.4 & 0.6\\ 0.6 & 0.4 \end{array}\right]$							
$(\alpha_{1},\alpha_{2},\phi ,\phi^{*},\lambda_{1}\left(1\right),\lambda_{1}\left(2\right),\lambda_{2}\left(1\right),\lambda_{2}\left(2\right))=\left(0.25,0.5,1,0.875,2,4,5,7\right)$							
Yule–Walker							
	$\alpha_{1}$	$\alpha_{2}$	ϕ	$\lambda_{1}\left(1\right)$	$\lambda_{1}\left(2\right)$	$\lambda_{2}\left(1\right)$	$\lambda_{2}\left(2\right)$
300	0.2424	0.4872	0.9863	1.9978	4.0018	4.9978	7.0024
sd	0.0716	0.0927	0.2995	0.1309	0.1858	0.2524	0.2967
bias	−0.0076	−0.0128	−0.0137	−0.0022	0.0018	−0.0022	0.0024
900	0.2479	0.4960	0.9954	2.0002	3.9995	5.0018	7.0022
sd	0.0414	0.0546	0.1732	0.0757	0.1067	0.1453	0.1713
bias	−0.0021	−0.0040	−0.0046	0.0002	−0.0005	0.0018	0.0022
1500	0.2489	0.4970	0.9967	1.9993	4.0005	4.9991	6.9997
sd	0.0317	0.0418	0.1332	0.0589	0.0818	0.1132	0.1347
bias	−0.0011	−0.0030	−0.0033	−0.0007	0.0005	−0.0009	−0.0003
2100	0.2495	0.4982	0.9992	1.9999	3.9999	4.9996	6.9995
sd	0.0271	0.0354	0.1139	0.0501	0.0696	0.0955	0.1138
bias	−0.0005	−0.0018	−0.0008	−0.0001	−0.0001	−0.0004	−0.0005
Conditional Maximum Likelihood							
	$\alpha_{1}$	$\alpha_{2}$	$\phi^{*}$	$\lambda_{1}\left(1\right)$	$\lambda_{1}\left(2\right)$	$\lambda_{2}\left(1\right)$	$\lambda_{2}\left(2\right)$
300	0.2508	0.4988	0.8848	1.9974	4.0012	4.9967	7.0017
sd	0.0447	0.0353	0.1692	0.1250	0.1760	0.2399	0.2843
bias	0.0008	−0.0012	0.0098	−0.0026	0.0012	−0.0033	0.0017
900	0.2503	0.4997	0.8791	1.9998	4.0019	4.9991	7.0012
sd	0.0245	0.0200	0.0937	0.0717	0.1024	0.1374	0.1640
bias	0.0003	−0.0003	0.0041	−0.0002	0.0019	−0.0009	0.0012
1500	0.2497	0.4993	0.8772	1.9987	4.0017	4.9964	6.9972
sd	0.0048	0.0126	0.0762	0.02969	0.0348	0.0097	0.0202
bias	−0.0002	−0.0006	0.0023	−0.0012	0.0017	−0.0035	−0.0028
2100	0.2502	0.4995	0.8769	1.9998	4.0002	4.9991	7.0001
sd	0.0023	0.0203	0.0105	0.0182	0.0215	0.0726	0.0499
bias	0.0002	−0.0005	0.0019	−0.0002	0.0002	−0.0009	0.0001

**Table A9:** Parameter estimation under two states; see Section 4.

Transition Matrix 2: $\left[\begin{array}{cc} 0.8 & 0.2\\ 0.2 & 0.8 \end{array}\right]$							
$(\alpha_{1},\alpha_{2},\phi ,\phi^{*},\lambda_{1}\left(1\right),\lambda_{1}\left(2\right),\lambda_{2}\left(1\right),\lambda_{2}\left(2\right))=\left(0.15,0.5,0.5,0.4625,1,3,3,5\right)$							
Yule–Walker							
	$\alpha_{1}$	$\alpha_{2}$	ϕ	$\lambda_{1}\left(1\right)$	$\lambda_{1}\left(2\right)$	$\lambda_{2}\left(1\right)$	$\lambda_{2}\left(2\right)$
300	0.1447	0.4824	0.4957	1.0008	2.9975	2.9954	4.9984
sd	0.0639	0.0923	0.1908	0.0937	0.1610	0.2206	0.2863
bias	−0.0053	−0.0176	−0.0042	0.0008	−0.0025	−0.0046	−0.0016
900	0.1472	0.4948	0.4984	0.9999	2.9986	2.9995	4.9985
sd	0.0384	0.0543	0.1104	0.0529	0.0922	0.1273	0.1643
bias	−0.0028	−0.0052	−0.0016	−0.0001	−0.0014	−0.0005	−0.0015
1500	0.1484	0.4966	0.4983	0.9996	3.0000	3.0018	5.0017
sd	0.0298	0.0417	0.0858	0.0412	0.0717	0.0981	0.1273
bias	−0.0016	−0.0034	−0.0016	−0.0004	0.0000	0.0018	0.0017
2100	0.1492	0.4974	0.4997	0.9996	3.0005	2.9983	5.0005
sd	0.0253	0.0354	0.0733	0.0348	0.0610	0.0835	0.1075
bias	−0.0008	−0.0026	−0.0002	−0.0004	0.0005	−0.0017	0.0005
Conditional Maximum Likelihood							
	$\alpha_{1}$	$\alpha_{2}$	$\phi^{*}$	$\lambda_{1}\left(1\right)$	$\lambda_{1}\left(2\right)$	$\lambda_{2}\left(1\right)$	$\lambda_{2}\left(2\right)$
300	0.1508	0.5156	0.4843	0.9962	3.0038	2.9873	5.0166
sd	0.0455	0.0371	0.0839	0.0843	0.1492	0.1870	0.2393
bias	0.0008	0.0156	0.0218	−0.0038	0.0038	−0.0127	0.0166
900	0.1492	0.5109	0.4720	0.9987	3.0032	2.9916	5.0075
sd	0.0243	0.0201	0.0462	0.0490	0.0858	0.1077	0.1385
bias	−0.0008	0.0109	0.0095	−0.0013	0.0032	−0.0084	0.0075
1500	0.1489	0.5091	0.4667	0.9991	3.0033	2.9939	5.0094
sd	0.0185	0.0162	0.0333	0.0380	0.0656	0.0831	0.1061
bias	−0.0011	0.0091	0.0042	−0.0009	0.0033	−0.0061	0.0094
2100	0.1488	0.5083	0.4651	0.9989	3.0012	2.9951	5.0066
sd	0.0154	0.0142	0.0266	0.0319	0.0566	0.0698	0.0896
bias	−0.0012	0.0083	0.0026	−0.0011	0.0012	−0.0049	0.0066

**Table A10:** Parameter estimation under two states; see Section 4.

Transition Matrix 2: $\left[\begin{array}{cc} 0.8 & 0.2\\ 0.2 & 0.8 \end{array}\right]$							
$(\alpha_{1},\alpha_{2},\phi ,\phi^{*},\lambda_{1}\left(1\right),\lambda_{1}\left(2\right),\lambda_{2}\left(1\right),\lambda_{2}\left(2\right))=\left(0.25,0.5,1,0.875,2,4,5,7\right)$							
Yule–Walker							
	$\alpha_{1}$	$\alpha_{2}$	ϕ	$\lambda_{1}\left(1\right)$	$\lambda_{1}\left(2\right)$	$\lambda_{2}\left(1\right)$	$\lambda_{2}\left(2\right)$
300	0.2401	0.4841	0.9838	2.0001	4.0000	4.9963	6.9980
sd	0.0691	0.0919	0.2969	0.1426	0.2023	0.2827	0.3425
bias	−0.0099	−0.0159	−0.0162	0.0001	0.0000	−0.0037	−0.0020
900	0.2463	0.4940	0.9940	1.9995	4.0002	4.9996	7.0014
sd	0.0399	0.0539	0.1718	0.0821	0.1138	0.1634	0.1942
bias	−0.0037	−0.0060	−0.0060	−0.0005	0.0002	−0.0004	0.0014
1500	0.2476	0.4964	0.9971	1.9996	4.0006	5.0001	7.0009
sd	0.0310	0.0413	0.1340	0.0633	0.0899	0.1278	0.1515
bias	−0.0024	−0.0036	−0.0029	−0.0004	0.0006	0.0001	0.0009
2100	0.2486	0.4977	0.9983	1.9999	3.9999	4.9991	6.9987
sd	0.0266	0.0353	0.1125	0.0534	0.0765	0.1069	0.1277
bias	−0.0014	−0.0023	−0.0017	−0.0001	−0.0001	−0.0009	−0.0013
Conditional Maximum Likelihood							
	$\alpha_{1}$	$\alpha_{2}$	$\phi^{*}$	$\lambda_{1}\left(1\right)$	$\lambda_{1}\left(2\right)$	$\lambda_{2}\left(1\right)$	$\lambda_{2}\left(2\right)$
300	0.2502	0.4997	0.8864	1.9974	4.0025	4.9994	7.0021
sd	0.0469	0.0362	0.1719	0.1271	0.1805	0.2406	0.2851
bias	0.0002	−0.0003	0.0114	−0.0026	0.0025	−0.0006	0.0021
900	0.2503	0.4997	0.8795	2.0002	3.9996	5.0024	7.0019
sd	0.0255	0.0203	0.0964	0.0733	0.1035	0.1389	0.1627
bias	0.0003	−0.0003	0.0045	0.0002	−0.0004	0.0024	0.0019
1500	0.2503	0.4995	0.8762	1.9994	4.0004	4.9998	6.9992
sd	0.0197	0.0157	0.0733	0.0566	0.0798	0.1078	0.1285
bias	0.0003	−0.0005	0.0012	−0.0006	0.0004	−0.0002	−0.0008
2100	0.2502	0.4997	0.8764	1.9998	4.0001	4.9993	6.9999
sd	0.0166	0.0133	0.0617	0.0485	0.0678	0.0907	0.1090
bias	0.0002	−0.0003	0.0014	−0.0002	0.0001	−0.0007	−0.0001

## Appendix B. Proof of Proposition 1

The bivariate PGF of $\left\{X_{1,t}\left(s_{t}\right),X_{2,t}\left(s_{t}\right)\right\}$ equals

$$\begin{array}{cc} E[a_{1}^{X_{1,t}\left(s_{t}\right)}\cdot a_{2}^{X_{2,t}\left(s_{t}\right)}]\; & =E[a_{1}^{\alpha_{1}\circ X_{1,t-1}\left(s_{t-1}\right)+\varepsilon_{1,t}\left(s_{t},s_{t-1}\right)}\cdot a_{2}^{\alpha_{2}\circ X_{2,t-1}\left(s_{t-1}\right)+\varepsilon_{2,t}\left(s_{t},s_{t-1}\right)}]\\ \; & =E[a_{1}^{\alpha_{1}\circ X_{1,t-1}\left(s_{t-1}\right)}\cdot a_{1}^{\varepsilon_{1,t}\left(s_{t},s_{t-1}\right)}\cdot a_{2}^{\alpha_{2}\circ X_{1,t-1}\left(s_{t-1}\right)}\cdot a_{2}^{\varepsilon_{2,t}\left(s_{t},s_{t-1}\right)}]\\ \; & =E[{\left(1-\alpha_{1}+\alpha_{1}a_{1}\right)}^{X_{1,t-1}\left(s_{t-1}\right)}\cdot {\left(1-\alpha_{2}+\alpha_{2}a_{2}\right)}^{X_{2,t-1}\left(s_{t-1}\right)}]\\ \; & \;\;\;\cdot E[a_{1}^{\varepsilon_{1,t}\left(s_{t},s_{t-1}\right)}\cdot a_{2}^{\varepsilon_{2,t}\left(s_{t},s_{t-1}\right)}]\\ \; & =\exp\{\lambda_{1}\left(s_{t-1}\right)\cdot \left(-\alpha_{1}+\alpha_{1}a_{1}\right)+\lambda_{2}\left(s_{t-1}\right)\cdot \left(-\alpha_{2}+\alpha_{2}a_{2}\right)\\ \; & \;\;\;+\phi \left(-\alpha_{1}+\alpha_{1}a_{1}\right)\left(-\alpha_{2}+\alpha_{2}a_{2}\right)\}\cdot E\left[a_{1}^{\varepsilon_{1,t}\left(s_{t},s_{t-1}\right)}\cdot a_{2}^{\varepsilon_{2,t}\left(s_{t},s_{t-1}\right)}\right]. \end{array}$$

According to the expression of the bivariate Poisson PGF, we know that

$$\begin{array}{c} E\left[a_{1}^{X_{1,t}\left(s_{t}\right)}\cdot a_{2}^{X_{2,t}\left(s_{t}\right)}\right]=\exp\left\{\lambda_{1}\left(s_{t}\right)\left(a_{1}-1\right)+\lambda_{2}\left(s_{t}\right)\left(a_{2}-1\right)+\phi \left(a_{1}-1\right)\left(a_{2}-1\right)\right\}. \end{array}$$

For simplicity, we omit the states index in the parentheses. Thus, the PGF of $\left\{\varepsilon_{1,t},\varepsilon_{2,t}\right\}$ is derived as

$$\begin{array}{cc} E\left[a_{1}^{\varepsilon_{1,t-1}}\cdot a_{2}^{\varepsilon_{2,t-1}}\right]=\exp\{ & \lambda_{1}\left(s_{t}\right)\left(a_{1}-1\right)+\lambda_{2}\left(s_{t}\right)\left(a_{2}-1\right)+\phi \left(a_{1}-1\right)\left(a_{2}-1\right)\\ \; & -\lambda_{1}\left(s_{t-1}\right)\left(-\alpha_{1}+\alpha_{1}a_{1}\right)-\lambda_{2}\left(s_{t-1}\right)\left(-\alpha_{2}+\alpha_{2}a_{2}\right)\\ & -\phi \left(-\alpha_{1}+\alpha_{1}a_{1}\right)\left(-\alpha_{2}+\alpha_{2}a_{2}\right)\}, \end{array}$$

which implies that the bivariate PGF of $\left\{\varepsilon_{1,t},\varepsilon_{2,t}\right\}$ is

$$\begin{array}{cc} E\left[a_{1}^{\varepsilon_{1,t-1}}\cdot a_{2}^{\varepsilon_{2,t-1}}\right]=\exp\{ & \left(\lambda_{1}\left(s_{t}\right)-\lambda_{1}\left(s_{t-1}\right)\cdot \alpha_{1}\right)\cdot \left(a_{1}-1\right)\\ \; & +\left(\lambda_{2}\left(s_{t}\right)-\lambda_{2}\left(s_{t-1}\right)\cdot \alpha_{2}\right)\cdot \left(a_{2}-1\right)\\ & +\left(\phi -\phi \cdot \alpha_{1}\cdot \alpha_{2}\right)\left(a_{1}-1\right)\left(a_{2}-1\right)\}. \end{array}$$

Thus, $\left\{\varepsilon_{1,t},\varepsilon_{2,t}\right\}∼\mathrm{BPoi}\left(\lambda_{1}\left(s_{t}\right)-\lambda_{1}\left(s_{t-1}\right)\alpha_{1},\;\lambda_{2}\left(s_{t}\right)-\lambda_{2}\left(s_{t-1}\right)\alpha_{2},\;\phi -\phi \alpha_{1}\alpha_{2}\right)$. Finally, with $\phi^{*}=\phi -\phi \alpha_{1}\alpha_{2}$, we write $\left\{\varepsilon_{1,t},\varepsilon_{2,t}\right\}∼\mathrm{BPoi}\left(\lambda_{1}\left(s_{t}\right)-\lambda_{1}\left(s_{t-1}\right)\alpha_{1},\lambda_{2}\left(s_{t}\right)-\lambda_{2}\left(s_{t-1}\right)\alpha_{2},\;\phi^{*}\right)$, which completes the proof of Proposition 1.

## Appendix C. Proof of Proposition 2

The considered (conditional) means are computed as follows:

$$\begin{array}{cc} E[X_{i,t}\left(s_{t}\right)|X_{i,t-1}\left(s_{t-1}\right)]\; & =E[\alpha_{i}\circ X_{i,t-1}\left(s_{t-1}\right)+\varepsilon_{i,t}\left(s_{t},s_{t-1}\right)|X_{i,t-1}\left(s_{t-1}\right)]\\ \; & =\alpha_{i}\cdot X_{i,t-1}\left(s_{t-1}\right)+\mu_{i}\left(s_{t},s_{t-1}\right), \end{array}$$

and

$$\begin{array}{cc} E[X_{i,t}\left(s_{t}\right)]\; & =E[E\left[X_{i,t}\left(s_{t}\right)|X_{i,t-1}\left(s_{t-1}\right)\right]]\\ \; & =E[E\left[\alpha_{i}\circ X_{i,t-1}\left(s_{t-1}\right)+\varepsilon_{i,t}\left(s_{t},s_{t-1}\right)|X_{i,t-1}\left(s_{t-1}\right)\right]]\\ \; & =E[\alpha_{i}\cdot X_{i,t-1}\left(s_{t-1}\right)+\mu_{i}\left(s_{t},s_{t-1}\right)]\\ \; & =\alpha_{i}\cdot E\left[X_{i,t-1}\left(s_{t-1}\right)\right]+\mu_{i}\left(s_{t},s_{t-1}\right)=\alpha_{i}\cdot \lambda_{i}\left(s_{t-1}\right)+\mu_{i}\left(s_{t},s_{t-1}\right). \end{array}$$

From the bivariate Poisson distribution of $\left\{\varepsilon_{1,t}\left(s_{t},s_{t-1}\right),\varepsilon_{2,t}\left(s_{t},s_{t-1}\right)\right\}$ according to Proposition 1, we have $\mu_{i}\left(s_{t},s_{t-1}\right)=\lambda_{i}\left(s_{t}\right)-\lambda_{i}\left(s_{t-1}\right)\cdot \alpha_{i}$. Thus,

$$\begin{array}{cc} E[X_{i,t}\left(s_{t}\right)] & =\alpha_{i}\cdot \lambda_{i}\left(s_{t-1}\right)+\lambda_{i}\left(s_{t}\right)-\lambda_{i}\left(s_{t-1}\right)\cdot \alpha_{i}=\lambda_{i}\left(s_{t}\right). \end{array}$$

From the Poisson’s equidispersion property, we obtain $Var\left(X_{i,t}\left(s_{t}\right)\right)=\lambda_{i}\left(s_{t}\right)$. This is used to compute

$$\begin{array}{cc} cov(X_{i,t}\left(s_{t}\right),X_{i,t-1}\left(s_{t-1}\right))\; & =cov(\alpha_{i}\circ X_{i,t-1}\left(s_{t-1}\right)+\varepsilon_{i,t}\left(s_{t},s_{t-1}\right),X_{i,t-1}\left(s_{t-1}\right))\\ \; & =cov(\alpha_{i}\circ X_{i,t-1}\left(s_{t-1}\right),X_{i,t-1}\left(s_{t-1}\right))\\ \; & \;\;\;\;\;+cov(X_{i,t-1}\left(s_{t-1}\right),\varepsilon_{i,t}\left(s_{t},s_{t-1}\right))\\ \; & =\alpha_{i}\cdot Var\left(X_{i,t-1}\left(s_{t-1}\right)\right)=\alpha_{i}\cdot \lambda_{i}\left(s_{t-1}\right). \end{array}$$

According to Proposition 1, we assume the marginal distribution of $\left\{X_{1,t}\left(s_{t}\right),X_{2,t}\left(s_{t}\right)\right\}$ to be $\mathrm{BPoi}(\lambda_{1}\left(s_{t}\right),\lambda_{2}\left(s_{t}\right),\phi )$, so

$$cov(X_{1,t}\left(s_{t}\right),X_{2,t}\left(s_{t}\right))=\phi .$$

Hence,

$$\begin{array}{cc} cov & \left(X_{1,t}\left(s_{t}\right),X_{2,t-k}\left(s_{t-k}\right)\right)=cov\left(\alpha_{1}^{k}\circ X_{1,t-k}\left(s_{t-k}\right)+\sum_{j=0}^{k-1}\alpha_{1}^{j}\circ \varepsilon_{t-j},X_{2,t-k}\left(s_{t-k}\right)\right)\\ \; & =cov\left(\alpha_{1}^{k}\circ X_{1,t-k}\left(s_{t-k}\right),X_{2,t-k}\left(s_{t-k}\right)\right)+cov\left(\sum_{j=0}^{k-1}\alpha_{1}^{j}\circ \varepsilon_{t-j},X_{2,t-k}\left(s_{t-k}\right)\right)\\ \; & =cov(\alpha_{1}^{k}\circ X_{1,t-k}\left(s_{t-k}\right),X_{2,t-k}\left(s_{t-k}\right))+0\\ \; & =\alpha_{1}^{k}\cdot cov\left(X_{1,t-k}\left(s_{t-k}\right),X_{2,t-k}\left(s_{t-k}\right)\right)=\alpha_{1}^{k}\cdot \phi . \end{array}$$

This completes the proof of Proposition 2.

## Appendix D. Proof of Remark 4

We rewrite $\phi =cov(X_{1,t}\left(s_{t}\right),X_{2,t}\left(s_{t}\right))$ as follows:

$$\begin{array}{cc} \; & cov\left(X_{1,t}\left(s_{t}\right),X_{2,t}\left(s_{t}\right)\right)=E\left[X_{1,t}\left(s_{t}\right)\cdot X_{2,t}\left(s_{t}\right)\right]-E\left[X_{1,t}\left(s_{t}\right)\right]\cdot E\left[X_{2,t}\left(s_{t}\right)\right]\\ \; & =\sum_{s=1}^{S}E\left[X_{1,t}\left(s_{t}\right)X_{2,t}\left(s_{t}\right)1_{\left\{s_{t}=s\right\}}\right]-\sum_{s=1}^{S}E\left[X_{1,t}\left(s_{t}\right)1_{\left\{s_{t}=s\right\}}\right]\cdot E\left[X_{2,t}\left(s_{t}\right)1_{\left\{s_{t}=s\right\}}\right]\\ \; & =\sum_{s=1}^{S}\left\{E\left[X_{1,t}\left(s\right)X_{2,t}\left(s\right)\right]1_{\left\{s_{t}=s\right\}}-E\left[X_{1,t}\left(s\right)\right]1_{\left\{s_{t}=s\right\}}\cdot E\left[X_{2,t}\left(s\right)\right]1_{\left\{s_{t}=s\right\}}\right\}\\ \; & =\sum_{s=1}^{S}\left\{E\left[X_{1,t}\left(s\right)X_{2,t}\left(s\right)\right]-E\left[X_{1,t}\left(s\right)\right]\cdot E\left[X_{2,t}\left(s\right)\right]\right\}1_{\left\{s_{t}=s\right\}}\\ \; & =\sum_{s=1}^{S}cov\left(X_{1,t}\left(s\right),X_{2,t}\left(s\right)\right)1_{\left\{s_{t}=s\right\}}=\sum_{s=1}^{S}cov\left(X_{1,t}\left(s_{t}\right),X_{2,t}\left(s_{t}\right)|s_{t}=s\right)1_{\left\{s_{t}=s\right\}}. \end{array}$$

Summing over *t* on both sides, we have

$$\begin{array}{cc} n\cdot \phi & =\sum_{t=1}^{n}cov\left(X_{1,t}\left(s_{t}\right),X_{2,t}\left(s_{t}\right)\right)=\sum_{t=1}^{n}\sum_{s=1}^{S}cov\left(X_{1,t}\left(s_{t}\right),X_{2,t}\left(s_{t}\right)|s_{t}=s\right)1_{\left\{s_{t}=s\right\}}\\ \; & =\sum_{t=1}^{n}\sum_{s=1}^{S}\gamma_{12,0}\left(s\right)1_{\left\{s_{t}=s\right\}}=\sum_{s=1}^{S}\sum_{t=1}^{n}\gamma_{12,0}\left(s\right)1_{\left\{s_{t}=s\right\}}=\sum_{s=1}^{S}n_{s}\cdot \gamma_{12,0}\left(s\right). \end{array}$$

Thus, $cov(X_{1,t}\left(s_{t}\right),X_{2,t}\left(s_{t}\right))$ can be expressed as as in Equation (8).

According to Proposition 2(ii), the $\alpha_{i}$ are expressed as follows:

$$\begin{array}{cc} \alpha_{i} & =\frac{cov(X_{i,t}\left(s_{t}\right),X_{i,t-1}\left(s_{t-1}\right))}{\lambda_{i}\left(s_{t-1}\right)}\\ \; & =\frac{\sum_{r=1}^{S}\sum_{s=1}^{S}cov\left(X_{i,t}\left(s_{t}\right),X_{i,t-1}\left(s_{t-1}\right)|s_{t-1}=r,s_{t}=s\right)1_{\left\{s_{t-1}=r,s_{t}=s\right\}}}{\sum_{r=1}^{S}\lambda_{i}\left(s_{t-1}\right)1_{\left\{s_{t-1}=r\right\}}}. \end{array}$$

Summing over *t* on both sides, we have

$$\begin{array}{cc} n\cdot \alpha_{i}=\sum_{t=1}^{n}\alpha_{i} & =\sum_{t=1}^{n}\frac{\sum_{r=1}^{S}\sum_{s=1}^{S}cov\left(X_{i,t}\left(s_{t}\right),X_{i,t-1}\left(s_{t-1}\right)|s_{t-1}=r,s_{t}=s\right)1_{\left\{s_{t-1}=r,s_{t}=s\right\}}}{\sum_{r=1}^{S}\lambda_{i}\left(s_{t-1}\right)1_{\left\{s_{t-1}=r\right\}}}\\ \; & =\sum_{t=1}^{n}\frac{\sum_{r=1}^{S}\sum_{s=1}^{S}\gamma_{i}\left(r,s\right)1_{\left\{s_{t}=s,s_{t-1}=r\right\}}}{\sum_{r=1}^{S}\gamma_{ii,0}\left(r\right)1_{\left\{s_{t-1}=r\right\}}}=\frac{\sum_{r=1}^{S}\sum_{s=1}^{S}n_{r,s}\gamma_{i}\left(r,s\right)}{\sum_{r=1}^{S}\gamma_{ii,0}\left(r\right)},\\ \mathrm{so}\;\alpha_{i} & =\sum_{r=1}^{S}\sum_{s=1}^{S}\frac{n_{r,s}}{n}\frac{\gamma_{i}\left(r,s\right)}{\gamma_{ii,0}\left(r\right)}. \end{array}$$

Hence, we obtain the expression in Equation (9), which completes the proof of Remark 4.

## Appendix E. Proof of Theorem 1

To prove the consistency of the YW estimators, we argue from the view of asymptotically uncorrelated sequences. According to Equations (5) and (6), ${\hat{\mu }}_{i}\left(s\right)$ only uses the sample collected under the same state *s*, which is $\left\{X_{i,t}\left(s\right)1_{\left\{s_{t}=s\right\}}\right\}$. For the corresponding sub-sample from $\left\{X_{i,t}\left(s_{t}\right)\right\}$, the correlation coefficient is

$$\rho_{\tau }=corr\left(X_{i,t-\tau }\left(s\right)1_{\left\{s_{t-\tau }=s\right\}},X_{i,t}\left(s\right)1_{\left\{s_{t}=s\right\}}\right)=\left\{\begin{array}{cc} \alpha_{i}^{\tau }, & \mathrm{if}\;s_{t-\tau }=s,s_{t}=s\\ 0, & \mathrm{otherwise}. \end{array}\right.$$

Thus,

$$\begin{array}{c} \sum_{\tau =0}^{\infty }\rho_{\tau }⩽\sum_{\tau =0}^{\infty }\alpha_{i}^{\tau }=\frac{1}{1-\alpha_{i}}. \end{array}$$

In addition to $\sum_{\tau =0}^{\infty }\rho_{\tau }<\infty$, we also know that $Var(X_{i,t}\left(s_{t}\right)|s_{t}=s)<\infty$ for all *t*. Thus, according to Definition 3.55 in [28], we know that $\left\{X_{i,t}\left(s\right)1_{\left\{s_{t}=s\right\}}\right\}$ is an asymptotically uncorrelated sequence.

Regarding the expression of ${\hat{\gamma }}_{i}\left(r,s\right)$, we obtain

$$\begin{array}{cc} {\hat{\gamma }}_{i}\left(r,s\right) & =\frac{1}{n_{r,s}}\sum_{t=2}^{n}\left(X_{i,t}\left(s_{t}\right)-{\hat{\mu }}_{i}\left(s\right)\right)\left(X_{i,t-1}\left(s_{t-1}\right)-{\hat{\mu }}_{i}\left(r\right)\right)1_{\left\{s_{t-1}=r,s_{t}=s\right\}}\\ \; & =\frac{1}{n_{r,s}}\sum_{t=2}^{n}X_{i,t}\left(s_{t}\right)X_{i,t-1}\left(s_{t-1}\right)1_{\left\{s_{t-1}=r,s_{t}=s\right\}}-\frac{1}{n_{r,s}}\sum_{t=2}^{n}X_{i,t}\left(s_{t}\right){\hat{\mu }}_{i}\left(r\right)1_{\left\{s_{t-1}=r,s_{t}=s\right\}}\\ \; & \;\;-\frac{1}{n_{r,s}}\sum_{t=2}^{n}X_{i,t-1}\left(s_{t-1}\right){\hat{\mu }}_{i}\left(s\right)1_{\left\{s_{t-1}=r,s_{t}=s\right\}}+\frac{1}{n_{r,s}}\sum_{t=2}^{n}{\hat{\mu }}_{i}\left(r\right){\hat{\mu }}_{i}\left(s\right)1_{\left\{s_{t-1}=r,s_{t}=s\right\}}\\ \; & =\frac{1}{n_{r,s}}\sum_{t=2}^{n}X_{i,t}\left(s_{t}\right)X_{i,t-1}\left(s_{t-1}\right)1_{\left\{s_{t-1}=r,s_{t}=s\right\}}-\frac{1}{n_{r,s}}{\hat{\mu }}_{i}\left(r\right)\sum_{t=2}^{n}X_{i,t}\left(s_{t}\right)1_{\left\{s_{t}=s\right\}}\\ \; & \;\;-\frac{1}{n_{r,s}}{\hat{\mu }}_{i}\left(s\right)\sum_{t=2}^{n}X_{i,t-1}\left(s_{t-1}\right)1_{\left\{s_{t-1}=r\right\}}+{\hat{\mu }}_{i}\left(r\right){\hat{\mu }}_{i}\left(s\right)\\ \; & =\frac{1}{n_{r,s}}\sum_{t=2}^{n}X_{i,t}\left(s_{t}\right)X_{i,t-1}\left(s_{t-1}\right)1_{\left\{s_{t-1}=r,s_{t}=s\right\}}-{\hat{\mu }}_{i}\left(r\right){\hat{\mu }}_{i}\left(s\right)-{\hat{\mu }}_{i}\left(r\right){\hat{\mu }}_{i}\left(s\right)+{\hat{\mu }}_{i}\left(r\right){\hat{\mu }}_{i}\left(s\right)\\ \; & =\frac{1}{n_{r,s}}\sum_{t=2}^{n}X_{i,t}\left(s_{t}\right)X_{i,t-1}\left(s_{t-1}\right)1_{\left\{s_{t-1}=r,s_{t}=s\right\}}-{\hat{\mu }}_{i}\left(r\right){\hat{\mu }}_{i}\left(s\right). \end{array}$$

Defining $Y_{i,t}=X_{i,t}\left(s_{t}\right)X_{i,t-1}\left(s_{t-1}\right)$, our next step is to derive an explicit expression for $E[Y_{i,t}\cdot Y_{i,t+\tau }]$. Here, we omit the term $\left(s_{t},s_{t-1}\right)$ after $\varepsilon_{i,t}$ for simplicity:

$$\begin{array}{cc} \; & \;E[Y_{i,t}\cdot Y_{i,t+\tau }]\\ \; & =E[X_{i,t-1}\left(s_{t-1}\right)\cdot X_{i,t}\left(s_{t}\right)\cdot X_{i,t+\tau }\left(s_{t+\tau }\right)\cdot X_{i,t+\tau -1}\left(s_{t+\tau -1}\right)]\\ \; & =E[X_{i,t-1}\left(s_{t-1}\right)\cdot X_{i,t}\left(s_{t}\right)\cdot (\alpha_{i}^{\tau }\circ X_{i,t}\left(s_{t}\right)\\ \; & +\sum_{j=0}^{\tau -1}\alpha_{i}^{j}\circ \varepsilon_{i,t+\tau -j})\cdot \left(\alpha_{i}^{\tau }\circ X_{i,t-1}\left(s_{t-1}\right)+\sum_{q=0}^{\tau -1}\alpha_{i}^{q}\circ \varepsilon_{i,t+\tau -1-q}\right)]\\ \; & =E[E[X_{i,t-1}\left(s_{t-1}\right)\cdot X_{i,t}\left(s_{t}\right)\cdot \left(\alpha_{i}^{\tau }\circ X_{i,t}\left(s_{t}\right)+\sum_{j=0}^{\tau -1}\alpha_{i}^{j}\circ \varepsilon_{i,t+\tau -j}\right)\\ \; & \cdot \left(\alpha_{i}^{\tau }\circ X_{i,t-1}\left(s_{t-1}\right)+\sum_{q=0}^{\tau -1}\alpha_{i}^{q}\circ \varepsilon_{i,t+\tau -1-q}\right)|F_{t}]]\\ \; & =E[X_{i,t-1}\left(s_{t-1}\right)\cdot X_{i,t}\left(s_{t}\right)\cdot E[\left(\alpha_{i}^{\tau }\circ X_{i,t}\left(s_{t}\right)+\sum_{j=0}^{\tau -1}\alpha_{i}^{j}\circ \varepsilon_{i,t+\tau -j}\right)\\ \; & \cdot \left(\alpha_{i}^{\tau }\circ X_{i,t-1}\left(s_{t-1}\right)+\sum_{q=0}^{\tau -1}\alpha_{i}^{q}\circ \varepsilon_{i,t+\tau -1-q}\right)|F_{t}]]\\ \; & =E[X_{i,t-1}\left(s_{t-1}\right)\cdot X_{i,t}\left(s_{t}\right)\cdot E[\alpha_{i}^{\tau }\circ X_{i,t}\left(s_{t}\right)\cdot \alpha_{i}^{\tau }\circ X_{i,t-1}\left(s_{t-1}\right)\\ \; & +\alpha_{i}^{\tau }\circ X_{i,t}\left(s_{t}\right)\cdot \left(\sum_{q=0}^{\tau -1}\alpha_{i}^{q}\circ \varepsilon_{i,t+\tau -1-q}\right)\\ \; & +\alpha_{i}^{\tau }\circ X_{i,t-1}\left(s_{t-1}\right)\cdot \left(\sum_{j=0}^{\tau -1}\alpha_{i}^{j}\circ \varepsilon_{i,t+\tau -j}\right)+\left(\sum_{j=0}^{\tau -1}\alpha_{i}^{j}\circ \varepsilon_{i,t+\tau -j}\right)\cdot \left(\sum_{q=0}^{\tau -1}\alpha_{i}^{q}\circ \varepsilon_{i,t+\tau -1-q}\right)|F_{t}]]\\ \; & =E[X_{i,t-1}\left(s_{t-1}\right)\cdot X_{i,t}\left(s_{t}\right)\cdot \alpha_{i}^{\tau }\cdot X_{i,t}\left(s_{t}\right)\cdot \alpha_{i}^{\tau }\cdot X_{i,t-1}\left(s_{t-1}\right)\\ \; & +X_{i,t-1}\left(s_{t-1}\right)\cdot X_{i,t}\left(s_{t}\right)\cdot \alpha_{i}^{\tau }\cdot X_{i,t}\left(s_{t}\right)\cdot \left(\sum_{q=0}^{\tau -1}\alpha_{i}^{q}\cdot E\left[\varepsilon_{i,t+\tau -1-q}\right]\right)\\ \; & +X_{i,t-1}\left(s_{t-1}\right)\cdot X_{i,t}\left(s_{t}\right)\cdot \alpha_{i}^{\tau }\cdot X_{i,t-1}\left(s_{t-1}\right)\cdot \left(\sum_{j=0}^{\tau -1}\alpha_{i}^{j}\cdot E\left[\varepsilon_{i,t+\tau -j}\right]\right)\\ \; & +X_{i,t-1}\left(s_{t-1}\right)\cdot X_{i,t}\left(s_{t}\right)\cdot \left(\sum_{j=0}^{\tau -1}\alpha_{i}^{j}\cdot E\left[\varepsilon_{i,t+\tau -j}\right]\right)\cdot \left(\sum_{q=0}^{\tau -1}\alpha_{i}^{q}\cdot E\left[\varepsilon_{i,t+\tau -1-q}\right]\right)]\\ \; & =\alpha_{i}^{2\tau }\cdot E\left[X_{i,t-1}^{2}\left(s_{t-1}\right)\cdot X_{i,t}^{2}\left(s_{t}\right)\right]\\ \; & +\alpha_{i}^{\tau }\cdot E\left[X_{i,t}^{2}\left(s_{t}\right)\cdot X_{i,t-1}\left(s_{t-1}\right)\right]\cdot \left(\sum_{q=0}^{\tau -1}\alpha_{i}^{q}\cdot E\left[\varepsilon_{i,t+\tau -1-q}\right]\right)\\ \; & +\alpha_{i}^{\tau }\cdot E\left[X_{i,t-1}^{2}\left(s_{t-1}\right)\cdot X_{i,t}\left(s_{t}\right)\right]\cdot \left(\sum_{j=0}^{\tau -1}\alpha_{i}^{j}\cdot E\left[\varepsilon_{i,t+\tau -j}\right]\right)\\ \; & +E\left[X_{i,t}\left(s_{t}\right)\cdot X_{i,t-1}\left(s_{t-1}\right)\right]\cdot \left(\sum_{q=0}^{\tau -1}\alpha_{i}^{q}\cdot E\left[\varepsilon_{i,t+\tau -1-q}\right]\right)\cdot \left(\sum_{j=0}^{\tau -1}\alpha_{i}^{j}\cdot E\left[\varepsilon_{i,t+\tau -j}\right]\right). \end{array}$$

As the Poisson distribution has existing moments, there are bounds $M_{4},M_{\varepsilon_{i}}$ such that $E\left[X_{i,t}^{4}\left(s_{t}\right)\right]<M_{4}<\infty$ and $E\left[\varepsilon_{i,t}\right]<M_{\varepsilon_{i}}=max\left\{\lambda_{i}\left(s\right),s=1,\ldots ,S\right\}$. Then, we have

$$\begin{array}{cc} \; & \sum_{j=0}^{\tau -1}\alpha_{i}^{j}\cdot E\left[\varepsilon_{i,t+\tau -j}\right]<M_{\varepsilon_{i}}\cdot \frac{\left(1-\alpha_{i}^{\tau -1}\right)}{1-\alpha_{i}},\\ & \sum_{q=0}^{\tau -1}\alpha_{i}^{q}\cdot E\left[\varepsilon_{i,t+\tau -1-q}\right]<M_{\varepsilon_{i}}\cdot \frac{\left(1-\alpha_{i}^{\tau -1}\right)}{1-\alpha_{i}}. \end{array}$$

According to the Hölder inequality, we obtain

$$\begin{array}{cc} \; & 0<E\left[X_{i,t-1}^{2}\left(s_{t-1}\right)\cdot X_{i,t}^{2}\left(s_{t}\right)\right]⩽{\left[E|X_{i,t-1}\left(s_{t-1}\right){|}^{4}\right]}^{\frac{1}{2}}\cdot {\left[E|X_{i,t}\left(s_{t}\right){|}^{4}\right]}^{\frac{1}{2}}⩽M_{4}^{4},\\ \; & 0<E\left[X_{i,t-1}^{2}\left(s_{t-1}\right)\cdot X_{i,t}\left(s_{t}\right)\right]⩽{\left[E|X_{i,t-1}\left(s_{t-1}\right){|}^{4}\right]}^{\frac{1}{2}}\cdot [E|X_{i,t}\left(s_{t}\right){|}^{2}{|}^{\frac{1}{2}}⩽M_{4}^{3},\\ \; & 0<E\left[X_{i,t-1}\left(s_{t-1}\right)\cdot X_{i,t}^{2}\left(s_{t}\right)\right]⩽{\left[E|X_{i,t-1}\left(s_{t-1}\right){|}^{2}\right]}^{\frac{1}{2}}\cdot {\left[E|X_{i,t}\left(s_{t}\right){|}^{4}\right]}^{\frac{1}{2}}⩽M_{4}^{3},\\ \; & 0<E\left[X_{i,t}\left(s_{t}\right)\cdot X_{i,t-1}\left(s_{t-1}\right)\right]⩽{\left[E|X_{i,t-1}\left(s_{t-1}\right){|}^{2}\right]}^{\frac{1}{2}}\cdot {\left[E|X_{i,t}\left(s_{t}\right){|}^{2}\right]}^{\frac{1}{2}}⩽M_{4}^{2}. \end{array}$$

In addition, $E\left[Y_{i,t}\right]=E\left[X_{i,t}\left(s_{t}\right)\cdot X_{i,t-1}\left(s_{t-1}\right)\right]$ and

$$\begin{array}{cc} E[Y_{i,t+\tau }] & =E\left[X_{i,t+\tau }\left(s_{t}\right)\cdot X_{i,t-1}\left(s_{t+\tau -1}\right)\right]\\ \; & =E[E\left[X_{i,t+\tau }\left(s_{t}\right)\cdot X_{i,t-1}\left(s_{t+\tau -1}\right)|F_{t}\right]]\\ \; & =E[E[\left(\alpha_{i}^{\tau }\circ X_{i,t}\left(s_{t}\right)+\sum_{j=0}^{\tau -1}\alpha_{i}^{j}\circ \varepsilon_{i,t+\tau -j}\right)\\ \; & \cdot \left(\alpha_{i}^{\tau }\circ X_{i,t-1}\left(s_{t-1}\right)+\sum_{q=0}^{\tau -1}\alpha_{i}^{q}\circ \varepsilon_{i,t+\tau -1-q}\right)|F_{t}]]\\ \; & =E[E[\alpha_{i}^{\tau }\circ X_{i,t}\left(s_{t}\right)\cdot \alpha_{i}^{\tau }\circ X_{i,t-1}\left(s_{t-1}\right)+\alpha_{i}^{\tau }\circ X_{i,t}\left(s_{t}\right)\cdot \left(\sum_{q=0}^{\tau -1}\alpha_{i}^{q}\circ \varepsilon_{i,t+\tau -1-q}\right)\\ \; & +\alpha_{i}^{\tau }\circ X_{i,t-1}\left(s_{t-1}\right)\cdot \left(\sum_{j=0}^{\tau -1}\alpha_{i}^{j}\circ \varepsilon_{i,t+\tau -j}\right)\\ \; & +\left(\sum_{j=0}^{\tau -1}\alpha_{i}^{j}\circ \varepsilon_{i,t+\tau -j}\right)\cdot \left(\sum_{q=0}^{\tau -1}\alpha_{i}^{q}\circ \varepsilon_{i,t+\tau -1-q}\right)|F_{t}]]\\ \; & =E[\alpha_{i}^{\tau }\cdot X_{i,t}\left(s_{t}\right)\cdot \alpha_{i}^{\tau }\cdot X_{i,t-1}\left(s_{t-1}\right)+\alpha_{i}^{\tau }\cdot X_{i,t}\left(s_{t}\right)\cdot \;\left(\sum_{q=0}^{\tau -1}\alpha_{i}^{q}\cdot E\left[\varepsilon_{i,t+\tau -1-q}\right]\right)\\ \; & +\alpha_{i}^{\tau }\cdot X_{i,t-1}\left(s_{t-1}\right)\cdot \left(\sum_{j=0}^{\tau -1}\alpha_{i}^{j}\cdot E\left[\varepsilon_{i,t+\tau -j}\right]\right)\\ \; & +\left(\sum_{j=0}^{\tau -1}\alpha_{i}^{j}\cdot E\left[\varepsilon_{i,t+\tau -j}\right]\right)\cdot \left(\sum_{q=0}^{\tau -1}\alpha_{i}^{q}\cdot E\left[\varepsilon_{i,t+\tau -1-q}\right]\right)]\\ \; & =\alpha_{i}^{2\tau }\cdot E\left[X_{i,t-1}\left(s_{t-1}\right)\cdot X_{i,t}\left(s_{t}\right)\right]+\alpha_{i}^{\tau }\cdot E\left[X_{i,t}\left(s_{t}\right)\right]\cdot \left(\sum_{q=0}^{\tau -1}\alpha_{i}^{q}\cdot E\left[\varepsilon_{i,t+\tau -1-q}\right]\right)\\ \; & +\alpha_{i}^{\tau }\cdot E\left[X_{i,t-1}\left(s_{t-1}\right)\right]\cdot \left(\sum_{j=0}^{\tau -1}\alpha_{i}^{j}\cdot E\left[\varepsilon_{i,t+\tau -j}\right]\right)\\ \; & +\left(\sum_{q=0}^{\tau -1}\alpha_{i}^{q}\cdot E\left[\varepsilon_{i,t+\tau -1-q}\right]\right)\cdot \left(\sum_{j=0}^{\tau -1}\alpha_{i}^{j}\cdot E\left[\varepsilon_{i,t+\tau -j}\right]\right) \end{array}$$

Then,

$$\begin{array}{cc} cov(Y_{i,t},Y_{i,t+\tau }) & =E\left[Y_{i,t}\cdot Y_{i,t+\tau }\right]-E\left[Y_{i,t}\right]\cdot E\left[Y_{i,t+\tau }\right]\\ \; & =\{\alpha_{i}^{2\tau }\cdot E\left[X_{i,t-1}^{2}\left(s_{t-1}\right)\cdot X_{i,t}^{2}\left(s_{t}\right)\right]\\ \; & +\alpha_{i}^{\tau }\cdot E\left[X_{i,t-1}^{2}\left(s_{t-1}\right)\cdot X_{i,t}\left(s_{t}\right)\right]\cdot \left(\sum_{q=0}^{\tau -1}\alpha_{i}^{q}\cdot E\left[\varepsilon_{i,t+\tau -1-q}\right]\right)\\ \; & +\alpha_{i}^{\tau }\cdot E\left[X_{i,t}^{2}\left(s_{t}\right)\cdot X_{i,t-1}\left(s_{t-1}\right)\right]\cdot \left(\sum_{j=0}^{\tau -1}\alpha_{i}^{j}\cdot E\left[\varepsilon_{i,t+\tau -j}\right]\right)\\ \; & +E\left[X_{i,t}\left(s_{t}\right)\cdot X_{i,t-1}\left(s_{t-1}\right)\right]\cdot \left(\sum_{q=0}^{\tau -1}\alpha_{i}^{q}\cdot E\left[\varepsilon_{i,t+\tau -1-q}\right]\right)\cdot \left(\sum_{j=0}^{\tau -1}\alpha_{i}^{j}\cdot E\left[\varepsilon_{i,t+\tau -j}\right]\right)\}\\ - & E\left[X_{i,t}\left(s_{t}\right)\cdot X_{i,t-1}\left(s_{t-1}\right)\right]\cdot \{\alpha_{i}^{2\tau }\cdot E\left[X_{i,t-1}\left(s_{t-1}\right)\cdot X_{i,t}\left(s_{t}\right)\right]\\ \; & +\alpha_{i}^{\tau }\cdot E\left[X_{i,t}\left(s_{t}\right)\right]\cdot \left(\sum_{q=0}^{\tau -1}\alpha_{i}^{q}\cdot E\left[\varepsilon_{i,t+\tau -1-q}\right]\right)\\ \; & +\alpha_{i}^{\tau }\cdot E\left[X_{i,t-1}\left(s_{t-1}\right)\right]\cdot \left(\sum_{j=0}^{\tau -1}\alpha_{i}^{j}\cdot E\left[\varepsilon_{i,t+\tau -j}\right]\right)\\ \; & +\left(\sum_{q=0}^{\tau -1}\alpha_{i}^{q}\cdot E\left[\varepsilon_{i,t+\tau -1-q}\right]\right)\cdot \left(\sum_{j=0}^{\tau -1}\alpha_{i}^{j}\cdot E\left[\varepsilon_{i,t+\tau -j}\right]\right)\}\\ \; & =\alpha_{i}^{2\tau }\cdot \left\{E\left[X_{i,t-1}^{2}\left(s_{t-1}\right)\cdot X_{i,t}^{2}\left(s_{t}\right)\right]-E{\left[X_{i,t-1}\left(s_{t-1}\right)\cdot X_{i,t}\left(s_{t}\right)\right]}^{2}\right\}\\ \; & +\alpha_{i}^{\tau }\cdot \left(\sum_{q=0}^{\tau -1}\alpha_{i}^{q}\cdot E\left[\varepsilon_{i,t+\tau -1-q}\right]\right)\{E\left[X_{i,t-1}^{2}\left(s_{t-1}\right)\cdot X_{i,t}\left(s_{t}\right)\right]-E\left[X_{i,t}\left(s_{t}\right)\right]\\ \; & \;\;\;\;\;\;\;\;\cdot E[X_{i,t-1}\left(s_{t-1}\right)\cdot X_{i,t}\left(s_{t}\right)]\}\\ \; & +\alpha_{i}^{\tau }\cdot \left(\sum_{j=0}^{\tau -1}\alpha_{i}^{j}\cdot E\left[\varepsilon_{i,t+\tau -j}\right]\right)\{E\left[X_{i,t}^{2}\left(s_{t}\right)\cdot X_{i,t-1}\left(s_{t-1}\right)\right]-E\left[X_{i,t-1}\left(s_{t-1}\right)\right]\\ \; & \;\;\;\;\;\;\;\;\cdot E[X_{i,t-1}\left(s_{t-1}\right)\cdot X_{i,t}\left(s_{t}\right)]\}\\ \; & <\alpha_{i}^{2\tau }\cdot E\left[X_{i,t-1}^{2}\left(s_{t-1}\right)\cdot X_{i,t}^{2}\left(s_{t}\right)\right]\\ \; & +\alpha_{i}^{\tau }\cdot M_{\varepsilon_{i}}\cdot \frac{\left(1-\alpha_{i}^{\tau -1}\right)}{1-\alpha_{i}}\cdot E\left[X_{i,t-1}^{2}\left(s_{t-1}\right)\cdot X_{i,t}\left(s_{t}\right)\right]\\ \; & +\alpha_{i}^{\tau }\cdot M_{\varepsilon_{i}}\cdot \frac{\left(1-\alpha_{i}^{\tau -1}\right)}{1-\alpha_{i}}\cdot E\left[X_{i,t-1}\left(s_{t-1}\right)\cdot X_{i,t}^{2}\left(s_{t}\right)\right]\\ \; & <\alpha_{i}^{\tau }\cdot M_{4}^{4}+\alpha_{i}^{\tau }\cdot M_{\varepsilon_{i}}\cdot \frac{\left(1-\alpha_{i}^{\tau -1}\right)}{1-\alpha_{i}}\cdot M_{4}^{3}+\alpha_{i}^{\tau }\cdot M_{\varepsilon_{i}}\cdot \frac{\left(1-\alpha_{i}^{\tau -1}\right)}{1-\alpha_{i}}\cdot M_{4}^{3}. \end{array}$$

Altogether, we have

$$\begin{array}{cc} cov(Y_{i,t},Y_{i,t+\tau }) & =E\left[Y_{i,t}\cdot Y_{i,t+\tau }\right]-E\left[Y_{i,t}\right]\cdot E\left[Y_{i,t+\tau }\right]\\ \; & <\alpha_{i}^{\tau }\cdot M_{4}^{4}+2\alpha_{i}^{\tau }\cdot M_{\varepsilon_{i}}\cdot \frac{\left(1-\alpha_{i}^{\tau -1}\right)}{1-\alpha_{i}}\cdot M_{4}^{3}<\alpha_{i}^{\tau }\cdot M_{4}^{4}+2\alpha_{i}^{\tau }\cdot M_{\varepsilon_{i}}\cdot M_{4}^{3}. \end{array}$$

Thus,

$$\begin{array}{cc} \sum_{\tau =0}^{\infty }\rho_{\tau }^{Y_{i}} & =\sum_{\tau =0}^{\infty }corr\left(Y_{i,t},Y_{i,t+\tau }\right)=\sum_{\tau =0}^{\infty }\frac{cov(Y_{i,t},Y_{i,t+\tau })}{\sqrt{Var(Y_{i,t})}\sqrt{Var(Y_{i,t+\tau })}}\\ \; & <\frac{\sum_{\tau =0}^{\infty }\left\{\alpha_{i}^{\tau }\cdot M_{4}^{4}+2\alpha_{i}^{\tau }\cdot M_{\varepsilon_{i}}\cdot M_{4}^{3}\right\}}{\sqrt{Var(Y_{i,t})}\sqrt{Var(Y_{i,t+\tau })}}=\frac{M_{4}^{4}\cdot \frac{1}{1-\alpha_{i}}+2M_{4}^{3}\cdot M_{\varepsilon_{i}}\cdot \frac{1}{1-\alpha_{i}}}{\sqrt{Var(Y_{i,t})}\sqrt{Var(Y_{i,t+\tau })}}\\ \; & =\frac{M_{4}^{3}\cdot \frac{1}{1-\alpha_{i}}\cdot \left(M_{4}^{4}+2M_{\varepsilon_{i}}\right)}{\sqrt{Var(Y_{i,t})}\sqrt{Var(Y_{i,t+\tau })}}<\infty . \end{array}$$

It is obvious that $Var(Y_{i,t})<\infty$ for all *t*. Hence, $\left\{Y_{i,t}\right\}$ are asymptotically uncorrelated sequences. Using Theorem 3.57 in [28], we have

$$\begin{array}{c} \frac{1}{n_{s}}\sum_{t=1}^{n}X_{i,t}\left(s_{t}\right)1_{\left\{s_{t}=s\right\}}\overset{d}{⟶}E\left[X_{i,t}\left(s_{t}\right)|s_{t}=s\right] \end{array}$$

which means that ${\hat{\mu }}_{i}\left(s\right)\overset{d}{⟶}\mu_{i}\left(s\right)$. Analogously,

$$\begin{array}{c} \frac{1}{n_{r,s}}\sum_{t=2}^{n}X_{i,t}\left(s_{t}\right)X_{i,t-1}\left(s_{t-1}\right)1_{\left\{s_{t-1}=r,s_{t}=s\right\}}\overset{d}{⟶}E\left[X_{i,t-1}\left(s_{t-1}\right)X_{i,t}\left(s_{t}\right)|s_{t-1}=r,s_{t}=s\right] \end{array}$$

which actually is ${\hat{\gamma }}_{i}\left(r,s\right)\overset{d}{⟶}\gamma_{i}\left(r,s\right)$. Thus, the consistency of the estimator ${\hat{\lambda }}_{i}{\left(s\right)}^{yw}$ can be proved. The way of getting ${\hat{\gamma }}_{ii,0}\left(r\right)\overset{d}{⟶}\gamma_{ii,0}\left(r\right)$ is similar to proof for ${\hat{\mu }}_{i}\left(s\right)\overset{d}{⟶}\mu_{i}\left(s\right)$, so we omit here.

According to the expression for $\alpha_{i}$ in Remark 4, together with Slutsky’s Theorem, the consistency of the estimators ${\hat{\alpha }}_{i}^{yw}$ and ${\hat{\phi }}^{yw}$ can be concluded, completing the proof of Theorem 1.
